# Supplementary material for: Uncertainty in the Timing of Origin of Animals and the Limits of Precision in Molecular Timescales
Source: Curr Biol. 2015 Nov 16;25(22):2939–50. doi: 10.1016/j.cub.2015.09.066 (PMC4651906; doi:10.1016/j.cub.2015.09.066)
Supplement: Document S1. Supplemental Experimental Procedures, Figures S1–S3, and Tables S1–S4 [file mmc1.pdf]

Current Biology

Supplemental Information

Uncertainty in the Timing of Origin of Animals and the Limits of Precision in Molecular Timescales

Mario dos Reis, Yuttapong Thawornwattana, Konstantinos Angelis, Maximilian J Telford, Philip C.J. Donoghue, and Ziheng Yang

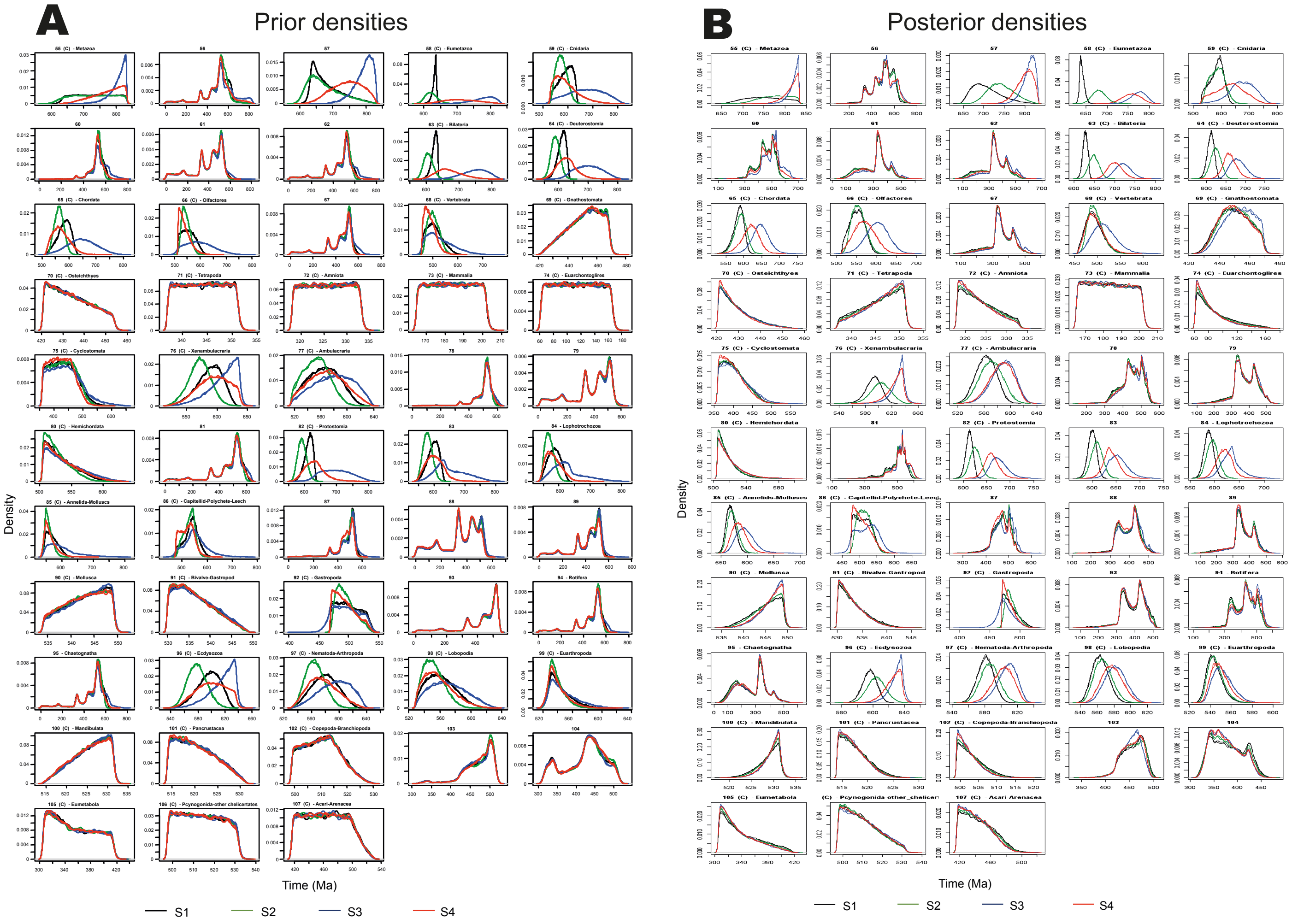

Figure S1.

Marginal prior (A) and marginal posterior (B) densities of divergence times for all nodes in the tree for the four different calibration strategies (Figure 1 in the main text). Nodes are numbered as in Figure 6 in the main text.

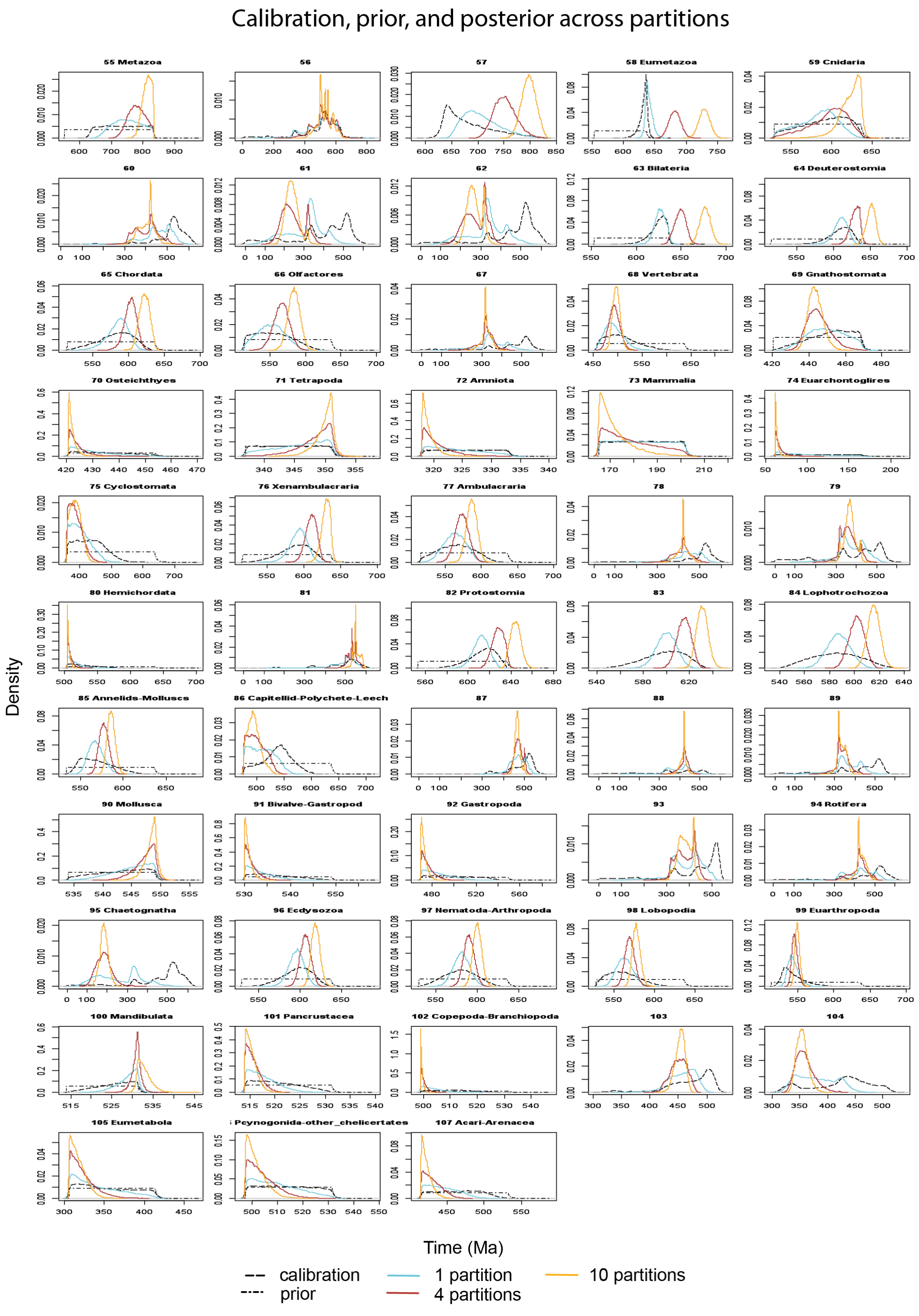

Figure S2.
Calibration, marginal prior and marginal posterior densities for various partitioning strategies. Nodes are numbered as in Figure 6 in the main text.

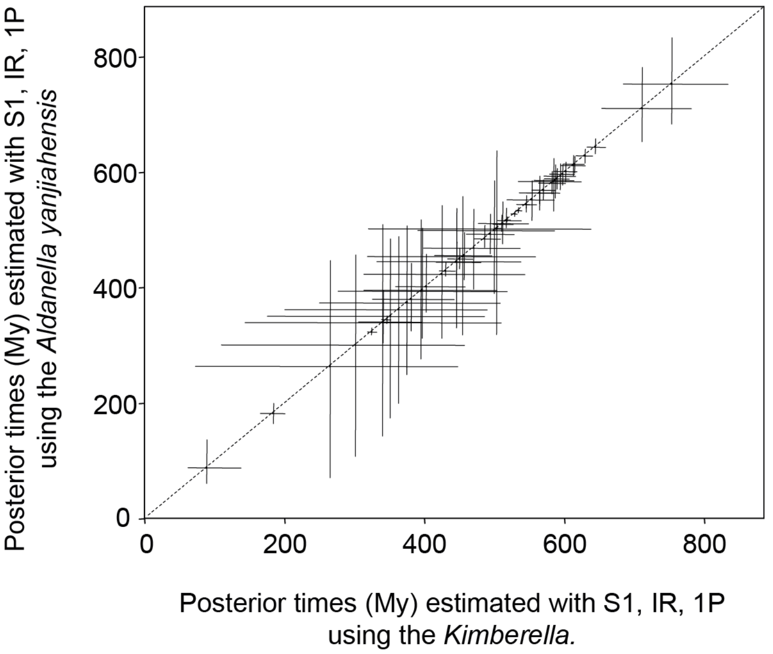

Figure S3.
Estimates of divergence times under calibration strategy 1, where the fossil *Kimberella* provides the minimum age constraint on the root, vs. estimates under strategy 1b, where *Aldanella* provides the minimum constraint instead. This extends Figure 2 in the main text.

Table S1. Fossil calibration densities constructed from the minimum and maximum contrains used in this study.

Node	Crown group	Min	Max	Strategy 1	Strategy 2	Strategy 3	Strategy 4
**55**	Metazoa	552.85	833	B(5.5285,8.33,0.001,0.001)	B(5.5285,8.33,0.001,0.001)	***B(6.349,8.33,0.001,0.001)***	***B(6.349,8.33,0.001,0.001)***
**58**	Eumetazoa	552.85	636.1	B(5.5285,6.361,0.001,0.025)	***SN(5.6,0.34,7)***	***L(5.5285,0,10,0.001)***	***L(5.5285,0,0.1,0.001)***
**59**	Cnidaria	529	636.1	B(5.29,6.361,0.001,0.025)	***SN(5.38,0.44,7)***	***L(5.29,0,10,0.001)***	***L(5.29,0,0.1,0.001)***
**63**	Bilateria	552.85	636.1	B(5.5285,6.361,0.001,0.025)	***SN(5.6,0.34,7)***	***L(5.5285,0,10,0.001)***	***L(5.5285,0,0.1,0.001)***
**64**	Deuterostomia	515.5	636.1	B(5.155,6.361,0.001,0.025)	***SN(5.255,0.5,7)***	***L(5.155,0,10,0.001)***	***L(5.155,0,0.1,0.001)***
**65**	Chordata	514	636.1	B(5.14,6.361,0.001,0.025)	***SN(5.25,0.5,7)***	***L(5.14,0,10,0.001)***	***L(5.14,0,0.1,0.001)***
**66**	Olfactores	514	636.1	B(5.14,6.361,0.001,0.025)	***SN(5.25,0.5,7)***	***L(5.14,0,10,0.001)***	***L(5.14,0,0.1,0.001)***
**68**	Vertebrata	457.5	636.1	B(4.575,6.361,0.001,0.025)	***SN(4.7,0.75,9)***	***L(4.575,0,10,0.001)***	***L(4.575,0,0.1,0.001)***
69	Gnathostomata	420.7	468.4	B(4.207,4.684,0.001,0.025)	B(4.207,4.684,0.001,0.025)	B(4.207,4.684,0.001,0.025)	B(4.207,4.684,0.001,0.025)
70	Osteichthyes	420.7	453.7	B(4.207,4.537,0.001,0.025)	B(4.207,4.537,0.001,0.025)	B(4.207,4.537,0.001,0.025)	B(4.207,4.537,0.001,0.025)
71	Tetrapoda	337	351	B(3.37,3.51,0.001,0.025)	B(3.37,3.51,0.001,0.025)	B(3.37,3.51,0.001,0.025)	B(3.37,3.51,0.001,0.025)
72	Amniota	318	332.9	B(3.18,3.329,0.001,0.025)	B(3.18,3.329,0.001,0.025)	B(3.18,3.329,0.001,0.025)	B(3.18,3.329,0.001,0.025)
73	Mammalia	164.9	201.5	B(1.649,2.015,0.001,0.025)	B(1.649,2.015,0.001,0.025)	B(1.649,2.015,0.001,0.025)	B(1.649,2.015,0.001,0.025)
74	Euarchontoglires	61.6	164.6	B(0.616,1.646,0.001,0.025)	B(0.616,1.646,0.001,0.025)	B(0.616,1.646,0.001,0.025)	B(0.616,1.646,0.001,0.025)
75	Cyclostomata	358.5	636.1	B(3.585,6.361,0.001,0.025)	B(3.585,6.361,0.001,0.025)	B(3.585,6.361,0.001,0.025)	B(3.585,6.361,0.001,0.025)
76	Xenambulacraria	515.5	636.1	B(5.155,6.361,0.001,0.025)	B(5.155,6.361,0.001,0.025)	B(5.155,6.361,0.001,0.025)	B(5.155,6.361,0.001,0.025)
77	Ambulacraria	515.5	636.1	B(5.155,6.361,0.001,0.025)	B(5.155,6.361,0.001,0.025)	B(5.155,6.361,0.001,0.025)	B(5.155,6.361,0.001,0.025)
80	Hemichordata	504.5	636.1	B(5.045,6.361,0.001,0.025)	B(5.045,6.361,0.001,0.025)	B(5.045,6.361,0.001,0.025)	B(5.045,6.361,0.001,0.025)
**82**	Protostomia	552.85	636.1	B(5.5285,6.361,0.001,0.025)	***SN(5.6,0.34,7)***	***L(5.5285,0,10,0.001)***	***L(5.5285,0,0.1,0.001)***
**85**	Annelids-Molluscs	534	636.1	B(5.34,6.361,0.001,0.025)	***SN(5.41,0.43,9)***	***L(5.34,0,10,0.001)***	***L(5.34,0,0.1,0.001)***
**86**	Capitellid-Polychete-leech	476.5	636.1	B(4.765,6.361,0.001,0.025)	***SN(4.86,0.68,10)***	***L(4.765,0,10,0.001)***	***L(4.765,0,0.1,0.001)***
90	Mollusca	534	549	B(5.34,5.49,0.001,0.025)	B(5.34,5.49,0.001,0.025)	B(5.34,5.49,0.001,0.025)	B(5.34,5.49,0.001,0.025)
91	Bivalve-Gastropod	530	549	B(5.30,5.49,0.001,0.025)	B(5.30,5.49,0.001,0.025)	B(5.30,5.49,0.001,0.025)	B(5.30,5.49,0.001,0.025)
**92**	Gastropoda	470.2	549	B(4.702,5.49,0.001,0.025)	***SN(4.75,0.33,9)***	***L(4.702,0,10,0.001)***	***L(4.702,0,0.1,0.001)***
96	Ecdysozoa	528.82	636.1	B(5.2882,6.361,0.001,0.025)	B(5.2882,6.361,0.001,0.025)	B(5.2882,6.361,0.001,0.025)	B(5.2882,6.361,0.001,0.025)

**97**	Nematoda-Arthropoda	528.82	636.1	B(5.2882,6.361,0.001,0.025)	***SN(5.38,0.44,7)***	***L(5.2882,0,10,0.001)***	***L(5.2882,0,0.1,0.001)***
98	Lobopodia	528.82	636.1	B(5.2882,6.361,0.001,0.025)	B(5.2882,6.361,0.001,0.025)	B(5.2882,6.361,0.001,0.025)	B(5.2882,6.361,0.001,0.025)
**99**	Euarthropoda	514	636.1	B(5.14,6.361,0.001,0.025)	***SN(5.22,0.52,9)***	***L(5.14,0,10,0.001)***	***L(5.14,0,0.1,0.001)***
100	Mandibulata	514	531.22	B(5.14,5.3122,0.001,0.025)	B(5.14,5.3122,0.001,0.025)	B(5.14,5.3122,0.001,0.025)	B(5.14,5.3122,0.001,0.025)
101	Pancrustacea	514	531.22	B(5.14,5.3122,0.001,0.025)	B(5.14,5.3122,0.001,0.025)	B(5.14,5.3122,0.001,0.025)	B(5.14,5.3122,0.001,0.025)
102	Copepoda-Branchiopoda	499	531.22	B(4.99,5.3122,0.001,0.025)	B(4.99,5.3122,0.001,0.025)	B(4.99,5.3122,0.001,0.025)	B(4.99,5.3122,0.001,0.025)
105	Eumetabola	305.5	413.6	B(3.055,4.136,0.001,0.025)	B(3.055,4.136,0.001,0.025)	B(3.055,4.136,0.001,0.025)	B(3.055,4.136,0.001,0.025)
106	Pycnogonida-other chelicertates	497.5	531.22	B(4.975,5.3122,0.001,0.025)	B(4.975,5.3122,0.001,0.025)	B(4.975,5.3122,0.001,0.025)	B(4.975,5.3122,0.001,0.025)
107	Acari-Arenacea	416	531.22	B(4.16,5.3122,0.001,0.025)	B(4.16,5.3122,0.001,0.025)	B(4.16,5.3122,0.001,0.025)	B(4.16,5.3122,0.001,0.025)

Note: B(t_L, t_U, p_L, p_U) means the node age has a soft uniform distribution between a minimum time t_L and a maximum time t_U, with probabilities p_L and p_U that the age is outside the bounds. SN(t, a, b) means the node age has a skew-normal distribution with location t, scale a, and shape b. L(t_L, p, c, p_L) means that the node age has a Cauchy distribution truncated on the left at t_L, with mode parameter p, tail parameter c, and probability p_L that the node age is younger than the minimum bound. Nodes and calibration densities that are different among the calibration strategies are indicated with bold typeface and italics. Fossil minima and maxima are from a draft version of Benton et al. (2015, *Paleontologica Electronica*, 18.1.1FC; 1–106). Note there are slight discrepancies between some of the minima and maxima above and the values in the final version of Benton et al. The discrepancies are around 2 My or less and are unimportant given the large width (max – min) of the calibrations. Node numbers as in Figure 6 in the main text.

Table S2. Minimum and maximum fossil constraints and 95% HPD CI of prior divergence times (Ma) for all metazoan clades under the four calibration strategies.

Node	Crown group	Min	Max	S1, IR, 1P		S2, IR, 1P		S3, IR, 1P		S4, IR, 1P	
55	Metazoa	552.85	833	641.3	832.6	629.5	833.2	757.5	833.5	689.0	833.3
56				123.1	679.0	115.6	664.5	160.0	826.9	129.8	768.3
57				622.4	778.9	599.9	777.8	738.2	832.5	662.6	826.7
58	Eumetazoa	552.85	636.1	616.6	642.7	589.5	658.7	717.4	830.5	629.4	806.7
59	Cnidaria	529	636.1	538.8	634.8	536.7	615.8	543.5	795.1	529.0	687.5
60				371.8	630.3	373.7	609.4	332.1	702.7	336.6	625.1
61				49.2	577.5	58.7	567.3	53.8	616.1	50.1	572.1
62				139.9	626.1	135.2	602.5	108.4	704.4	121.3	631.0
63	Bilateria	552.85	636.1	605.4	637.6	579.6	635.6	677.0	817.9	598.3	751.5
64	Deuterostomia	515.5	636.1	581.0	633.7	558.2	618.9	618.4	785.4	564.5	695.3
65	Chordata	514	636.1	535.2	623.1	532.1	596.3	546.0	749.5	519.0	630.1
66	Olfactores	514	636.1	513.9	598.1	518.1	576.0	506.8	696.9	513.9	584.2
67				107.8	592.6	112.6	573.8	110.6	641.8	89.7	570.1
68	Vertebrata	457.5	636.1	457.4	565.4	464.6	545.7	451.9	633.4	457.4	532.4
69	Gnathostomata	420.7	468.4	429.6	469.6	430.1	470.1	429.6	469.6	429.8	469.5
70	Osteichthyes	420.7	453.7	420.7	451.2	420.7	451.4	420.7	451.2	420.6	451.2
71	Tetrapoda	337	351	337.2	350.9	337.2	350.9	337.2	350.9	337.4	351.0
72	Amniota	318	332.9	318.3	332.9	318.2	332.7	318.3	332.8	318.1	332.6
73	Mammalia	164.9	201.5	165.6	201.2	165.5	201.2	165.9	201.5	165.6	201.2
74	Euarchontoglires	61.6	164.6	63.2	163.6	63.9	163.8	63.5	163.6	63.3	163.5
75	Cyclostomata	358.5	636.1	358.1	509.9	358.3	500.6	358.1	539.8	358.2	491.4
76	Xenambulacraria	515.5	636.1	547.0	625.3	534.4	605.8	561.6	643.2	548.2	638.8
77	Ambulacraria	515.5	636.1	519.3	605.8	516.1	586.7	526.8	630.5	517.6	616.2
78				335.9	592.6	338.9	578.2	331.8	607.7	334.7	595.2
79				45.3	556.6	51.0	552.9	34.7	550.4	50.1	556.5
80	Hemichordata	504.5	636.1	504.2	577.4	504.2	563.8	504.3	593.7	504.3	584.4
81				133.2	613.3	136.6	595.9	140.3	634.7	146.0	628.0
82	Protostomia	552.85	636.1	587.2	634.4	567.3	619.5	620.8	786.0	573.6	693.7
83				563.3	628.8	552.0	608.3	572.3	756.5	551.3	655.7
84				548.9	619.4	545.1	597.7	549.2	726.2	543.4	631.5
85	Annelids-Molluscs	534	636.1	539.4	605.5	539.8	582.6	539.6	693.8	536.4	601.8
86	Capitellid-Polychete-Leech	476.5	636.1	476.7	581.5	487.2	566.3	471.7	642.7	476.5	565.5
87				321.4	570.8	323.4	557.4	321.0	607.6	315.4	559.4
88				39.9	542.9	41.3	538.2	37.3	545.9	37.1	534.8
89				85.5	567.6	90.9	557.9	70.6	591.6	82.0	558.7
90	Mollusca	534	549	535.2	549.3	535.2	549.3	535.4	549.5	535.0	549.2
91	Bivalve-Gastropod	530	549	530.0	545.2	530.0	544.7	530.0	545.3	530.0	544.9
92	Gastropoda	470.2	549	470.2	532.7	472.3	527.0	460.7	536.8	470.1	528.2
93				106.6	544.9	106.7	545.5	100.6	544.4	108.4	545.5
94				134.0	613.7	134.2	592.0	104.0	646.5	126.1	612.9
95				147.7	623.1	140.4	600.8	110.5	685.0	137.9	634.0
96	Ecdysozoa	528.82	636.1	562.8	627.6	551.4	607.6	575.8	641.6	560.3	638.2
97	Nematoda-Arthropoda	528.82	636.1	543.0	614.7	539.7	591.8	551.7	634.0	538.2	617.8
98	Lobopodia	528.82	636.1	529.7	595.0	529.3	577.1	531.7	613.4	528.8	594.2
99	Euarthropoda	514	636.1	520.8	574.9	522.3	560.6	521.3	588.1	519.5	567.8
100	Mandibulata	514	531.22	517.6	532.0	517.6	531.8	517.6	531.9	517.3	531.7
101	Pancrustacea	514	531.22	514.0	528.2	514.0	528.1	514.0	528.3	514.0	528.0
102	Copepoda-	499	531.22	499.0	522.3	499.0	522.1	499.0	522.4	499.0	522.0

	Branchiopoda										
103				388.4	524.7	393.1	525.3	386.4	524.9	396.9	524.7
104				321.7	507.1	323.4	509.4	319.4	505.1	322.4	506.8
105	Eumetabola	305.5	413.6	305.4	409.2	305.4	409.7	305.5	409.6	305.4	409.1
106	Pcynogonida-other chelicertates	497.5	531.22	497.5	529.9	497.5	529.8	497.6	530.1	497.5	529.7
107	Acari-Arenacea	416	531.22	415.9	509.7	416.0	509.8	416.0	509.3	416.0	509.6

Note: Prior times are 95% HPD intervals estimated by running MCMCTree without sequence data under the four calibration strategies. S1–S4. IR: Independent-rates model. 1P: The 203 proteins analysed as a single partition. Node numbers are as in Figure 6 in the main text.

Table S3. Minimum and maximum fossil constraints and 95% HPD CI of posterior divergence times (Ma) for all metazoan clades under the four calibration strategies.

Node	Crown group	Min	Max	S1, IR, 1P		S2, IR, 1P		S3, IR, 1P		S4, IR, 1P	
55	Metazoa	552.85	833	680.6	832.7	716.2	833.4	795.2	833.6	780.0	833.5
56				314.6	639.9	318.6	646.6	319.2	670.6	319.2	661.1
57				649.2	776.7	686.1	805.5	779.5	832.2	761.6	831.4
58	Eumetazoa	552.85	636.1	630.7	652.9	649.5	714.2	738.5	808.8	715.4	798.7
59	Cnidaria	529	636.1	533.3	620.5	537.7	631.9	583.8	760.0	531.5	715.7
60				318.9	554.4	319.3	550.0	350.7	637.9	319.6	591.2
61				110.4	458.5	118.1	452.7	126.1	475.4	129.2	461.8
62				125.5	488.1	133.8	485.5	188.3	542.2	167.2	519.5
63	Bilateria	552.85	636.1	615.1	637.8	624.2	672.3	685.4	759.2	666.4	736.4
64	Deuterostomia	515.5	636.1	593.7	627.9	598.0	649.6	643.7	721.7	625.9	695.3
65	Chordata	514	636.1	555.4	611.3	558.1	622.2	600.5	693.3	568.6	662.6
66	Olfactores	514	636.1	516.6	583.6	524.3	588.0	548.2	656.1	521.8	618.6
67				167.9	480.9	193.2	485.3	236.2	526.3	203.9	486.4
68	Vertebrata	457.5	636.1	459.6	527.9	467.1	527.6	469.2	564.7	461.8	533.5
69	Gnathostomata	420.7	468.4	432.9	468.7	433.9	468.6	435.9	469.4	433.8	468.4
70	Osteichthyes	420.7	453.7	420.6	444.1	420.6	443.9	420.6	443.6	420.6	441.9
71	Tetrapoda	337	351	338.3	351.4	338.4	351.5	338.8	351.6	338.7	351.6
72	Amniota	318	332.9	318.0	331.4	318.0	331.1	318.0	330.7	318.0	330.7
73	Mammalia	164.9	201.5	165.1	200.7	164.9	200.5	164.9	200.6	165.0	200.5
74	Euarchontoglires	61.6	164.6	61.4	140.2	61.4	135.3	61.4	127.6	61.3	128.4
75	Cyclostomata	358.5	636.1	358.1	458.0	358.1	455.8	358.1	469.1	358.1	453.0
76	Xenambulacraria	515.5	636.1	569.8	614.5	575.9	632.2	606.4	646.4	600.6	644.4
77	Ambulacraria	515.5	636.1	534.6	591.3	538.5	603.5	554.8	620.1	552.7	618.8
78				330.6	537.8	334.3	541.3	348.9	550.0	343.3	548.1
79				250.6	507.0	266.4	509.1	285.6	510.8	277.5	508.1
80	Hemichordata	504.5	636.1	504.2	537.6	504.2	540.0	504.1	545.6	504.2	546.2
81				378.5	585.8	404.9	594.0	421.7	605.3	420.1	605.0
82	Protostomia	552.85	636.1	598.0	626.4	603.6	647.5	644.4	712.3	632.2	690.5
83				582.7	616.2	587.6	633.1	620.3	693.2	610.6	672.4
84				570.0	605.7	573.7	618.3	596.5	671.0	588.8	649.1
85	Annelids-Molluscs	534	636.1	552.3	586.1	554.1	591.7	564.2	630.1	559.5	611.9
86	Capitellid-Polychete-Leech	476.5	636.1	476.3	548.1	480.9	550.9	468.3	573.6	476.4	550.0
87				398.5	536.0	407.4	534.2	413.1	548.3	406.2	533.6
88				310.5	501.1	312.3	489.6	315.3	499.9	312.1	481.2
89				201.6	487.0	220.6	485.2	231.3	482.9	226.7	473.5
90	Mollusca	534	549	538.4	549.6	539.1	549.7	540.8	550.0	540.5	550.0
91	Bivalve-	530	549	530.0	539.1	530.0	538.6	530.0	538.2	530.0	538.3

92	Gastropod Gastropoda	470.2	549	470.0	508.3	470.3	506.2	450.8	505.3	470.1	500.9
93				265.0	516.5	285.1	512.1	300.3	505.4	291.9	507.6
94				310.6	541.4	314.2	538.2	319.4	549.4	318.5	544.6
95				72.7	452.4	84.3	447.5	93.3	454.7	88.8	447.7
96	Ecdysozoa	528.82	636.1	577.8	613.2	581.9	627.1	610.1	644.5	602.6	641.6
97	Nematoda-Arthropoda	528.82	636.1	561.4	599.8	563.8	608.3	583.9	628.6	577.8	625.0
98	Lobopodia	528.82	636.1	545.1	582.8	547.8	588.5	558.5	606.1	554.7	602.0
99	Euarthropoda	514	636.1	530.8	559.4	531.9	560.7	535.4	571.0	534.5	567.1
100	Mandibulata	514	531.22	523.4	532.3	524.0	532.3	525.2	532.6	525.0	532.4
101	Pancrustacea	514	531.22	514.0	522.8	514.0	522.3	514.0	521.8	514.0	521.9
102	Copepoda-Branchiopoda	499	531.22	499.0	510.1	498.9	509.2	498.9	508.0	498.9	508.3
103				414.4	496.1	414.2	493.6	418.0	490.3	417.6	491.5
104				324.8	441.5	325.3	438.8	327.0	433.4	327.6	435.5
105	Eumetabola	305.5	413.6	305.3	396.8	305.3	393.1	305.2	387.2	305.2	388.3
106	Pcynogonida-other chelicertates	497.5	531.22	497.5	526.1	497.5	525.8	497.5	526.9	497.5	526.4
107	Acari-Arenacea	416	531.22	415.9	479.9	415.8	477.5	415.9	474.0	415.8	474.4

Note: Posterior times are the 95% HPD interval, estimated with MCMCTree under the LG+G_4+F model, using four calibration strategies S1–S4. IR: Independent-rates model. 1P: The 203 proteins analysed as a single partition. Node numbers are as in Figure 6 in the main text.

Table S4. 95% HPD CI of posterior divergence times (Ma) for all metazoan clades under various partitioning strategies.

Node	Crown group	S1, IR, 1P		S2, IR, 2P		S3, IR, 4P		S4, IR, 5P		S4, IR, 10P	
55	Metazoa	680.6	832.7	701.0	831.2	736.9	832.6	748.9	832.3	786.8	833.5
56		314.6	639.9	326.2	632.6	387.9	639.0	413.3	639.2	440.3	631.0
57		649.2	776.7	674.7	781.6	712.5	794.3	726.8	798.5	771.7	823.1
58	Eumetazoa	630.7	652.9	638.9	669.0	664.2	699.9	677.5	711.0	712.2	746.2
59	Cnidaria	533.3	620.5	532.7	620.2	548.7	635.6	559.0	637.4	596.2	641.7
60		318.9	554.4	315.9	531.3	315.7	501.8	310.6	487.4	335.4	469.1
61		110.4	458.5	119.5	388.5	154.1	349.4	159.3	336.3	186.2	320.4
62		125.5	488.1	155.3	432.4	176.7	365.6	187.8	358.0	207.0	331.1
63	Bilateria	615.1	637.8	623.1	643.3	636.6	660.0	646.4	666.5	665.6	688.3
64	Deuterostomia	593.7	627.9	602.2	630.1	617.0	640.5	624.2	644.0	639.5	662.3
65	Chordata	555.4	611.3	567.9	611.3	586.2	619.3	593.4	621.6	609.0	635.7
66	Olfactores	516.6	583.6	527.9	584.5	544.0	586.5	552.0	589.6	568.0	600.0
67		167.9	480.9	215.4	444.5	233.7	391.3	246.4	383.9	274.6	371.0
68	Vertebrata	459.6	527.9	464.5	520.8	472.3	515.6	475.4	514.4	483.3	512.9
69	Gnathostomata	432.9	468.7	432.5	464.7	433.6	457.4	434.8	456.2	436.2	451.3
70	Osteichthyes	420.6	444.1	420.6	437.8	420.6	430.6	420.6	428.8	420.6	425.0
71	Tetrapoda	338.3	351.4	339.8	351.7	342.7	351.9	343.8	352.0	346.5	352.1
72	Amniota	318.0	331.4	318.0	329.3	318.0	325.2	318.0	323.9	318.0	321.5
73	Mammalia	165.1	200.7	164.9	200.0	164.8	197.8	164.8	196.4	164.8	186.5
74	Euarchontoglires	61.4	140.2	61.3	102.8	61.3	76.8	61.3	73.2	61.3	67.3
75	Cyclostomata	358.1	458.0	358.3	442.2	358.1	426.1	358.2	420.4	358.3	416.5
76	Xenambulacraria	569.8	614.5	580.7	615.2	595.1	623.7	601.7	626.6	617.6	639.9

77	Ambulacraria	534.6	591.3	542.9	588.2	555.1	591.4	559.6	592.3	572.6	600.1
78		330.6	537.8	331.9	516.0	341.2	488.9	348.9	481.5	367.8	469.9
79		250.6	507.0	268.9	468.2	296.2	445.1	304.2	436.2	317.9	422.4
80	Hemichordata	504.2	537.6	504.1	525.8	504.1	517.5	504.1	515.5	504.1	511.4
81		378.5	585.8	441.9	575.7	492.5	578.8	497.3	576.4	526.9	588.8
82	Protostomia	598.0	626.4	605.5	628.4	617.6	637.8	624.1	640.3	635.3	653.5
83		582.7	616.2	591.8	618.1	603.6	626.5	609.7	628.6	621.2	640.5
84		570.0	605.7	578.4	605.9	590.2	613.5	595.6	615.6	605.7	625.4
85	Annelids-Molluscs	552.3	586.1	559.2	585.3	567.2	588.8	570.6	590.3	577.4	595.1
86	Capitellid-Polychete-Leech	476.3	548.1	476.3	536.4	476.3	528.3	476.3	526.4	476.3	517.5
87		398.5	536.0	421.0	517.4	435.6	507.6	439.3	505.6	439.2	493.5
88		310.5	501.1	320.7	469.9	362.9	465.2	371.9	462.2	384.0	446.6
89		201.6	487.0	248.1	452.6	265.9	417.3	272.5	401.6	295.2	379.2
90	Mollusca	538.4	549.6	540.8	549.8	543.4	549.9	544.3	550.0	545.8	550.3
91	Bivalve-Gastropod	530.0	539.1	530.0	536.5	530.0	534.2	530.0	533.7	530.0	532.6
92	Gastropoda	470.0	508.3	470.1	497.9	470.0	487.4	470.0	484.6	470.0	478.8
93		265.0	516.5	304.5	486.8	313.2	452.3	314.3	444.6	324.8	431.8
94		310.6	541.4	317.1	512.1	344.0	502.9	349.3	489.6	394.2	481.2
95		72.7	452.4	90.5	350.9	110.4	254.0	122.5	248.1	140.6	225.2
96	Ecdysozoa	577.8	613.2	585.3	613.2	594.3	618.4	599.6	620.5	608.8	628.9
97	Nematoda-Arthropoda	561.4	599.8	568.6	598.4	577.7	602.4	581.6	604.0	589.8	610.4
98	Lobopodia	545.1	582.8	551.8	580.3	558.4	581.2	561.8	582.9	568.5	587.0
99	Euarthropoda	530.8	559.4	534.8	555.5	538.9	554.5	540.1	554.3	543.3	556.2
100	Mandibulata	523.4	532.3	526.2	532.6	528.4	533.1	528.9	533.4	530.3	536.1
101	Pancrustacea	514.0	522.8	514.0	520.4	514.0	518.5	514.0	518.0	514.0	517.5
102	Copepoda-Branchiopoda	499.0	510.1	498.9	505.8	498.9	502.5	498.9	501.8	498.9	500.5
103		414.4	496.1	420.1	485.7	423.7	476.8	426.1	474.9	435.2	468.1
104		324.8	441.5	328.0	424.0	330.4	392.4	332.8	387.2	334.8	374.4
105	Eumetabola	305.3	396.8	305.3	378.9	305.2	352.7	305.3	347.2	305.3	335.8
106	Pcynogonida-other chelicertates	497.5	526.1	497.4	520.5	497.4	514.7	497.4	512.5	497.4	509.1
107	Acari-Arenacea	415.9	479.9	415.9	466.3	415.8	453.2	415.7	448.4	415.8	436.4

Note: Posterior times are the 95% HPD interval, estimated with MCMCTree v4.8 under the LG+G_4+F model, using calibration strategies 1 and different partition strategies. 1P: The 203 proteins analysed as a single partition. 2P, 4P, 5P , 10P: The proteins are grouped into 2, 4, 5, 10 partitions according to their evolutionary rates. Node numbers are as in Figure 6 in the main text.

Supplemental Experimental Procedures

Data assembly

Two independent molecular data sets from [1] and [2] were updated and combined into a single amino acid alignment. Missing or incomplete proteins in the original alignments were updated with the non-redundant protein database from GenBank, with priority given to proteins from RefSeq. The protein sequence of each gene in each original alignment was used as a query for the BLASTp program. The best hit was accepted if the e-value was below 10^{-30} and the mismatches (excluding gaps) were lower than 5%. In addition, 5 new species (*Homo sapiens*, *Mus musculus*, *Ornithorhynchus anatinus*, *Tribolium castaneum* and *Caenorhabditis elegans*) were added to the data set in order to accommodate more calibration points. This was done in a similar manner as described above, but with the sequence of a closely related species according to [1] as a query instead.

For each gene, amino acid sequences of all species were aligned with PRANK [3]. The alignment gaps were removed using GBLOCKS [4] with the same setting as in [1]. All sub-alignments of individual genes were combined into a single alignment. This alignment contains 71 species with 38,577 amino acid positions from 203 nuclear coding genes (missing data 21.49%). This process recovered the original alignments but with extra species and sequences of genes previously missing or incomplete. Note that one gene was present in both data sets [1, 2], and hence was removed before the two data sets were combined. Further modifications to this alignment are described below.

Tree topology

The tree topology used is mainly according to [1] with some adjustments based on current knowledge. As the relationships among many taxa remain largely unresolved, 17 species were removed from the dataset in order to reduce the uncertainty in the topology. This resulted in a smaller alignment of the remaining 54 species (missing data 13.97%). The tree topology for these 54 species has 4 uncertain nodes that can be rearranged in three ways and one uncertain node that can be rearranged in two ways, giving $3^4 \times 2 = 162$ possible fully resolved trees. One of these trees was chosen (based on generally accepted positions) for the main analysis while the other 161 trees were used to assess the robustness of the time estimates to the various topologies.

Data partitioning

Two partitioning schemes were considered. First, the relative evolutionary rates among genes (evolutionary distances) were used for partitioning. Amino acid distance estimates for each gene were obtained from pairwise comparisons between *Strongylocentrotus purpuratus* and *Hydra magnipapillata* under the WAG+Γ_4+F model in CODEML v4.5 [5]. These two species were chosen because of their deep divergence time and because they have the most complete sequence data. For one missing gene of *Strongylocentrotus purpuratus*, the same gene of *Saccoglossus kowalevskii*, its close relative, was used instead. Because the divergence time is the same for all genes, the estimated distances can be used as a measure of the evolutionary rate. These distances were used to assign the 203 genes into different rate groups. Here, the numbers of rate categories (hence the number of partitions) considered were two, four, five, ten, as well as a single partition.

There is a possibility, however, that the rate estimated from these two chosen species may not be representative of the rates across branches, or the rates could be too varied to be used for partitioning. To address this issue, the suitability of this partitioning scheme was assessed by calculating the branch lengths of each partition using the WAG+Γ_4+F model. If the use of the rates is suitable in partitioning the data, the sum of the branch lengths (i.e. tree length) is expected to be approximately ordered from a partition with the lowest rate

category to one with the highest rate category. This was found to be the case.

Second, the data was divided into two partitions according to hydrophobicity, using the hydropathy index [6]. An average of the hydropathy index for each site in the alignment was calculated (gaps excluded). Then the site was classified as hydrophilic if the averaged hydropathy index was negative, otherwise it was classified as hydrophobic. Then the times were estimated with these two partitions under the LG+Γ_4+F model and again under the GTR+Γ_4+F model. For both models, the partitioning was virtually the same as the two-partition analysis according to rate and thus are not reported here.

Fossil Constraints

Thirty-four calibrations were derived mainly from ref [7] with updates from refs [8, 9]. The minimum ages were determined from the oldest certain record belonging to one of the two sister clades. These inferred minima are conservative and the actual origination time of a clade is likely to be older. The maximum ages were derived from the base of the youngest stratigraphic range or geological formation known not to contain any members of the clade of interest [10, 11]. Note that inferring a maximum date involves higher uncertainty and in most cases, the true origination dates are expected to be closer to the minimum constraint. A critical fossil is the Ediacaran *Kimberella* (552.85 Ma) which we interpret as a protostome, thus providing the minimum age constraint for Metazoa, Eumetazoa, Bilateria and Protostomia.

Fossil calibrations must be represented as statistical distributions mapped onto nodes. The choice of statistical distribution is subjective and may have a strong impact on estimated times [8, 12]. To assess the robustness of Metazoan divergences to calibration choice, we used four calibration strategies (Table S1):

(1) Strategy 1 (S1): The 34 calibrations are represented as uniform distributions between the min. and max. bounds. Bounds are soft, and we assign 0.1% and 2.5% tail probabilities that min. and max. bounds are violated (but we use 0.1% for both min. and max. bounds on the age of the root). A variation of S1 was also tested were the Cambrian snail *Aldanella* (532 Ma) was used instead of Kimberella to constrain the basal nodes. This change did not affect the results significantly.

(2) Strategy 2 (S2): 13 calibrations are represented as skewed-normal distributions. This was done for nodes for which the oldest ingroup fossil is thought to be very close to the actual parent node being calibrated. The parameters of the skew-normal (location, scale, shape) were thus chosen to provide a distribution with the mode near the minimum bound and the tail extending towards old ages, with the 0.3% and 97.5% quantiles of the distribution lying roughly at the equivalent minimum and maximum bounds from strategy 1. The remaining 21 nodes are as in strategy 1.

(3) Strategy 3 (S3): The same 13 nodes are calibrated using a truncated Cauchy distribution [12] with 0.1% left tail probability, with the mode of the distribution on the minimum bound, and with tail parameter equal to 10, leading to a long right tail for the distribution. No maximum bound is imposed on these nodes. The root node has an older minimum bound (634.9 Ma) accounting for alternative fossil interpretations.

(4) Strategy 4 (S4): Like strategy 3, but the tail parameter is 0.1 rather than 10, producing a truncated-Cauchy calibration with a much shorter tail. Note that the Cauchy is a heavy-tail distribution, and the tail thus extend further back in time than in the case of the skew-normal (S2).

Divergence time estimation

All molecular dating analyses were performed using the program MCMCtree in the PAML v4.8 package [5]. Analysis details are as follows:

(1) Priors for times

The time unit was set to 100 My. The prior on times was constructed using the fossil calibrations and the birth-death process [13]. The parameters of the birth-death process were set to λ = μ = 1, ρ = 0. This gives a uniform kernel and hence represents a diffuse prior.

In theory, one should specify a joint prior distribution of all node ages (and of all rates) in a Bayesian dating analysis, which should summarize all information in the fossil record. However, specifying high-dimensional prior densities with strong correlation structures is a task too daunting. Instead, current dating programs allow the user to specify calibration densities on individual node ages, and then a truncation is applied to ensure that ancestral nodes are older than descendant nodes. In MCMCTREE, this truncated joint density is combined with the probability density for the non-calibrated nodes specified by the birth-death process to produce the effective prior, i.e. the prior used by the program. As a result, a marginal prior distribution used in MCMCTREE can be very different from the specified calibration density. To assess the truncation on marginal distributions in the marginal prior were obtained by running the MCMC without sequence data and compared with the calibration densities. In addition, comparing these marginal priors with the marginal posteriors for times allows the relative impact of the prior and the sequence information to be assessed. The marginal priors for all the nodes are shown in Figure S1A.

(2) Approximate likelihood calculation

Since the data set used in this study is large, the likelihood function for each data partition was calculated using the approximate likelihood method [14, 15]. First, the branch lengths were estimated in CODEML using the LG+Γ_4+F model [16, 17]. Next, the estimated branch lengths, the gradient and the Hessian matrix were used to compute the likelihood by the second-order Taylor approximation to the likelihood function [15]. This method greatly reduces the computational time in likelihood calculation compared to the exact method, which could take much longer for a data set of this size.

(3) Rate drift model and priors on rates

We used the independent rate model, which assumes that the rates for all branches are i.i.d. log-normal random variables [18]. The log-normal density is specified by two parameters, the mean of the rate μ (representing the overall rate) and the variance σ^2 of the log-rate (determining the degree of rate variation across branches). Here, the gamma-Dirichlet prior was used for both parameters [19]. The overall rate, μ, was assigned a gamma prior, G(2, 40), which is a diffuse prior with a mean of 0.05 (meaning 5×10^{-10} amino acid substitutions per site per year). This was derived from the average pairwise amino acid distances between the 203 proteins of *Hydra magnipapillata* and *Strongylocentrotus purpuratus* (0.29 substitutions/site) assuming a divergence time of 636.1 Ma, so that the mean rate is 0.29/6.361 = 0.46 ≈ 0.05. By fixing the shape parameter to 2, this gives the scale parameter as 40. The prior for σ^2 was set to G(1, 10). We also used the autocorrelated-rates model [18] to assess the effect of rate-drift model on time estimates. The same gamma priors on μ was used and the prior on σ^2 was set to G(1, 10).

The number of iterations, the burn-in and the sampling frequency were adjusted in test runs of the program. The step sizes of the proposals used in MCMC were adjusted such that the acceptance proportions were close to 0.3. In addition, at least two chains were run to ensure convergence. Convergence was assessed by comparing the posterior means and plotting the time series traces of the MCMC samples from the two independent runs. The

resulting posterior distribution from one of the two runs was summarized and the means and 95% HPD intervals were collected.

Data availability

The molecular sequence alignment and trees with fossil calibrations have been deposited in Figshare: DOI: 10.6084/m9.figshare.1525089.

References

S1. Philippe, H., Brinkmann, H., Copley, R.R., Moroz, L.L., Nakano, H., Poustka, A.J., Wallberg, A., Peterson, K.J., and Telford, M.J. (2011). Acoelomorph flatworms are deuterostomes related to Xenoturbella. Nature *470*, 255-225.

S2. Erwin, D.H., Laflamme, M., Tweedt, S.M., Sperling, E.A., Pisani, D., and Peterson, K.J. (2011). The Cambrian Conundrum: Early Divergence and Later Ecological Success in the Early History of Animals. Science *334*, 1091-1097.

S3. Löytynoja, A., and Goldman, N. (2005). An algorithm for progressive multiple alignment of sequences with insertions. Proc Natl Acad Sci USA *102*, 10557-10562.

S4. Castresana, J. (2000). Selection of conserved blocks from multiple alignments for their use in phylogenetic analysis. Molecular biology and evolution *17*, 540-552.

S5. Yang, Z. (2007). PAML 4: Phylogenetic analysis by maximum likelihood. Mol. Biol. Evol. *24*, 1586-1591.

S6. Kyte, J., and Doolittle, R.F. (1982). A simple method for displaying the hydropathic character of a protein. Journal of molecular biology *157*, 105-132.

S7. Benton, M.J., Donoghue, P.C.J., and Asher, R.J. (2009). Calibrating and constraining molecular clocks. In The Timetree of Life, B.S. Hedges and S. Kumar, eds. (Oxford, England: Oxford University Press), pp. 35-86.

S8. Warnock, R.C., Yang, Z., and Donoghue, P.C. (2012). Exploring uncertainty in the calibration of the molecular clock. Biology letters *8*, 156-159.

S9. Benton, M.J., Donoghue, P.C.J., Asher, R.J., Friedman, M., Near, T.J., and Vinther, J. (2015). Constraints on the timescale of animal evolutionary history. Paleontolog. Elect.

S10. Benton, M.J., and Donoghue, P.C.J. (2007). Paleontological evidence to date the tree of life. Mol. Biol. Evol. *24*, 26-53.

S11. Donoghue, P.C., and Benton, M.J. (2007). Rocks and clocks: calibrating the tree of life using fossils and molecules. Trends Ecol. Evol. *22*, 424-431.

S12. Inoue, J., Donoghue, P.C.H., and Yang, Z. (2010). The impact of the representation of fossil calibrations on Bayesian estimation of species divergence times. Syst. Biol. *59*, 74-89.

S13. Yang, Z., and Rannala, B. (2006). Bayesian estimation of species divergence times under a molecular clock using multiple fossil calibrations with soft bounds. Mol. Biol. Evol. *23*, 212-226.

S14. Thorne, J.L., Kishino, H., and Painter, I.S. (1998). Estimating the rate of evolution of the rate of molecular evolution. Mol. Biol. Evol. *15*, 1647-1657.

S15. dos Reis, M., and Yang, Z. (2011). Approximate likelihood calculation for Bayesian estimation of divergence times. Mol. Biol. Evol. *28*, 2161–2172.

S16. Le, S.Q., and Gascuel, O. (2008). An improved general amino acid replacement matrix. Mol. Biol. Evol. *25*, 1307-1320.

S17. Yang, Z. (1994). Maximum likelihood phylogenetic estimation from DNA sequences with variable rates over sites: approximate methods. J. Mol. Evol. *39*, 306-314.

S18. Rannala, B., and Yang, Z. (2007). Inferring speciation times under an episodic molecular clock. Syst. Biol. *56*, 453-466.
S19. dos Reis, M., Zhu, T., and Yang, Z. (2014). The impact of the rate prior on Bayesian estimation of divergence times with multiple Loci. Syst. Biol. *63*, 555-565.
