# Supplementary material for: Uncertainty in the Timing of Origin of Animals and the Limits of Precision in Molecular Timescales
Source: Curr Biol. 2015 Nov 16;25(22):2939–50. doi: 10.1016/j.cub.2015.09.066 (PMC4651906; doi:10.1016/j.cub.2015.09.066)
Supplement: Document S2. Article plus Supplemental Information [file mmc2.pdf]

Article

Current Biology

Uncertainty in the Timing of Origin of Animals and the Limits of Precision in Molecular Timescales

Highlights

- Molecular clock analysis indicates an ancient origin of animals in the Cryogenian
- Diversification into animal phyla occurred in the Ediacaran, before the Cambrian
- Uncertainties in the fossil record and the molecular clock affect time estimates
- A precise timeline of animal evolution cannot be obtained with current methods

Authors

Mario dos Reis, Yuttapong Thawornwattana, Konstantinos Angelis, Maximilian J. Telford, Philip C.J. Donoghue, Ziheng Yang

Correspondence

phil.donoghue@bristol.ac.uk (P.C.J.D.), z.yang@ucl.ac.uk (Z.Y.)

In Brief

dos Reis et al. study the uncertainty in Bayesian estimates of the time of origin of animals using phylogenomic data. They find that disentangling the timeline of early animal evolution is hard given the large uncertainty associated with the fossil record of early animals and the strong violation of the molecular clock in ancient phylogenies.

CrossMark

dos Reis et al., 2015, Current Biology *25*, 2939–2950
November 16, 2015 ©2015 The Authors
http://dx.doi.org/10.1016/j.cub.2015.09.066

CellPress

Current Biology
Article

Uncertainty in the Timing of Origin of Animals and the Limits of Precision in Molecular Timescales

Mario dos Reis,[1,2] Yuttapong Thawornwattana,[1] Konstantinos Angelis,[1] Maximilian J. Telford,[1] Philip C.J. Donoghue,[3,*] and Ziheng Yang[1,*]
[1]Department of Genetics, Evolution, and Environment, University College London, Gower Street, London WC1E 6BT, UK
[2]School of Biological and Chemical Sciences, Queen Mary University of London, Mile End Road, London E1 4NS, UK
[3]School of Earth Sciences, University of Bristol, Life Sciences Building, Tyndall Avenue, Bristol BS8 1TQ, UK
*Correspondence: phil.donoghue@bristol.ac.uk (P.C.J.D.), z.yang@ucl.ac.uk (Z.Y.)
http://dx.doi.org/10.1016/j.cub.2015.09.066
This is an open access article under the CC BY license (http://creativecommons.org/licenses/by/4.0/).

SUMMARY

The timing of divergences among metazoan lineages is integral to understanding the processes of animal evolution, placing the biological events of species divergences into the correct geological timeframe. Recent fossil discoveries and molecular clock dating studies have suggested a divergence of bilaterian phyla >100 million years before the Cambrian, when the first definite crown-bilaterian fossils occur. Most previous molecular clock dating studies, however, have suffered from limited data and biases in methodologies, and virtually all have failed to acknowledge the large uncertainties associated with the fossil record of early animals, leading to inconsistent estimates among studies. Here we use an unprecedented amount of molecular data, combined with four fossil calibration strategies (reflecting disparate and controversial interpretations of the metazoan fossil record) to obtain Bayesian estimates of metazoan divergence times. Our results indicate that the uncertain nature of ancient fossils and violations of the molecular clock impose a limit on the precision that can be achieved in estimates of ancient molecular timescales. For example, although we can assert that crown Metazoa originated during the Cryogenian (with most crown-bilaterian phyla diversifying during the Ediacaran), it is not possible with current data to pinpoint the divergence events with sufficient accuracy to test for correlations between geological and biological events in the history of animals. Although a Cryogenian origin of crown Metazoa agrees with current geological interpretations, the divergence dates of the bilaterians remain controversial. Thus, attempts to build evolutionary narratives of early animal evolution based on molecular clock timescales appear to be premature.

INTRODUCTION

The timing and tempo of the evolutionary emergence of animal biodiversity has been among the most enduring problems in evolutionary biology. Innumerable hypotheses have been proposed to explain how the transition to multicellularity was effected, why it occurred when it did, and why it did not occur much earlier in Earth history [1]. Much of the molecular genetic toolkit required for animal development originated deep in eukaryote evolutionary history [2], and it has been widely held that the emergence of complex multicellular organisms was precluded until the oxygenation of the biosphere [3, 4]. Other potential, but not necessarily mutually exclusive, triggers for animal diversification include the release of their forebears from the environmental strictures of the Cryogenian or Ediacaran Snowball Earth [5, 6] and the effects of cosmic radiation [7], polar wander [8], continental fragmentation [9], H_2S toxicity [10], salinity [11, 12], a scarcity of trace metal micronutrients [13], a pulse of continental weathering yielding nutrients to the oceans [14], global warming [15], or an escalatory predator-prey arms race [16, 17]. Although these hypotheses propose more or less proximal causal mechanistic relationships with metazoan diversification, they rely ultimately on presumed temporal coincidence. This is challenging since the timing and the threshold of extrinsic environmental effects is invariably as unclear as the timing of the phenomena that they have been invoked to explain (e.g., [18]), varying from the origin of animals, eumetazoans, or bilaterians, to the origin of the animal phyla themselves, their crown radiations, or the sum total of this diversity. Indeed, it has been argued that the oxygenation of global oceans was a consequence, not a cause, of metazoan evolution [19]. Reconciling these competing hypotheses requires calibration to a common absolute timescale.

Unequivocal fossil evidence of animals is limited to the Phanerozoic. Older records of animals are controversial: organic biomarkers indicative of demosponges [20] are apparently derived ultimately from now symbiotic bacteria [21]; putative animal embryo fossils [22] are alternately interpreted as protists [23–25]; and contested reports of sponges [26, 27], molluscs [28], and innumerable cnidarians [29], as well as putative traces of eumetazoan or bilaterian grade animals [30–34], all from the Ediacaran. Certainly, there are no unequivocal records of crown-group bilaterians prior to the Cambrian [35], and robust evidence for bilaterian phyla does not occur until some 20 million years into the Cambrian [36, 37]. There is, nevertheless, increasingly general acceptance of a Precambrian history to animal evolution, and it is only its extent that remains open to debate. Was there an explosive radiation of bilaterian phyla close to the base of Cambrian [36, 38, 39]? Or is there an extensive Precambrian bilaterian

history that extends deep into the Cryogenian [35], the absence of a fossil record merely reflecting preservation, collection, and/or interpretation biases?

It has been hoped that these questions may be answered, and a timescale for animal evolutionary history established, using molecular clock methodology. Indeed, there is a long history of attempts to estimate the timing of animal diversification [40], yielding ages for crown Metazoa that range between 1,298 Ma [41] and 615 Ma [42]. The disparity between molecular clock estimates and fossil evidence of clade age minima has diminished in association with the development of molecular clock methodology, particularly in accommodating rate variation. Molecular clock timescales are considered good enough by some to begin to synthesize evolutionary narratives integrating evidence of extrinsic environmental and ecological evolution from the geological record with intrinsic biological evolution [43, 44]. Most recent divergence time analyses have been undertaken within a framework of Bayesian inference because it is capable of integrating much of the uncertainty associated with divergence time estimation, viz. the relationships between fossil evidence and clade age, rate variation among lineages (the relaxed clock), branch length estimation, tree topology, and parameters such as data partitioning. Yet, few studies have considered the cumulative impact of these uncertainties on the precision of evolutionary timescales (e.g., [45, 46]).

Here we show that the precision of molecular clock estimates of times has been grossly over-estimated. Using a Bayesian method to estimate the timescale of metazoan diversification, we performed sensitivity analyses to explore the impact of the different sources of uncertainties. We used a large amino acid alignment (38,577 sites) of 203 nuclear encoded proteins for 71 species (based on [35, 47]). We employed four fossil calibration strategies that accommodate different interpretations of the fossil record and show that these have a dramatic impact on the estimated times. We also explored the use of different relaxed clock models and show that at this level of divergence the molecular clock is significantly violated. We tested for the effects of different data partitioning strategies and show that this, too, has a significant impact on divergence time estimates. Finally, we show that competing phylogenetic hypotheses yield very different divergence time estimates. An evolutionary timescale for metazoan diversification that accommodates these uncertainties has precision that is insufficient to discriminate among causal hypotheses. Though some of this uncertainty can be reduced through increased precision of calibrations afforded by statistical modeling of fossil occurrence, more sequence data, reduced topological uncertainty, etc., the limitations of the fossil record and the confounding effect of times and rates will remain, making it difficult to achieve the precision required to test competing hypotheses on the causes and consequences of metazoan diversification.

RESULTS

The Impact of Uncertainty in Fossil Calibrations

Fossil calibrations are affected by numerous sources of uncertainty, including phylogenetic interpretation, dating of the rocks, and estimates of the time gap between the fossil minimum and the true clade ages [48]. This uncertainty is accommodated by statistical distributions describing the prior paleontological estimates of the true node ages within a phylogeny. Since a node cannot be older than its ancestors, the specified calibration densities are truncated to accommodate this intrinsic constraint from tree topology, generating the effective prior used by the dating program. The resulting marginal prior densities on clade ages can be quite different from the fossil calibration densities specified originally [49].

To assess the robustness of estimated Metazoan divergences to calibration choice, we established temporal constraints on the ages of 34 clades based on fossil evidence (Table 1). These were used as the basis for four competing sets of calibration densities, reflecting different interpretations of the fossil evidence (Table S1). Under strategies 1 and 2, the age of crown Metazoa has the minimum constraint based on a protostome interpretation of the Ediacaran *Kimberella*, whereas in strategies 3 and 4 it is based on the disputed biogeochemical evidence of Cryogenian demosponges [20, 27]. In strategy 1, all 34 calibrations were modeled as uniform distributions with soft bounds [50]. In strategies 2–4, we used different calibration densities for 14 phylum and superphylum crown nodes. In strategy 2, these 14 nodes are modeled using a skew-normal distribution with the mode of the distribution near the minimum bound and the tail extending into the past. These calibrations represent an optimistic interpretation of the fossil minima as a close approximation of the true clade age. In strategies 3 and 4, the 14 nodes use truncated Cauchy distributions [51] with either a long tail (strategy 3) or a short tail (strategy 4), extending back in time. This represents a pessimistic interpretation of palaeontological evidence in which the first fossil records of clades are a poor approximation of their antiquity. Note that the Cauchy is a heavy-tailed distribution, that is, it places considerable probability mass on its tail (contrary to the skew normal, which is light tailed). The calibration-based time prior is shown in Table S2.

The program MCMCTree [52] was used to obtain posterior time estimates under these four strategies and on the fixed tree topology of Figure 1. The evolutionary rates on branches of the tree were assumed to vary independently among lineages (the independent rates [IR] model [53]). All gene alignments were concatenated and analyzed as a single partition (1P) under the LG + Γ amino acid substitution model. In all instances, we first ran the analyses without sequence data to establish the effective time prior. This allowed us to evaluate the impact of truncation, which can yield marginal time priors that differ considerably from the original fossil evidence [49, 51].

Calibration strategy has a large impact on estimated divergence times (Figure 1A, Table S3, and Figure S1). Estimates under strategy 1 indicate that Metazoa originated 833–681 Ma, Bilateria 638–615 Ma, Deuterostomia 628–594 Ma, and Protostomia 626–598 Ma (Table S3). When the skew-normal distribution is employed (which places the majority of its probability mass near the minimum age bound; strategy 2), the resulting posterior time estimates agree largely with those obtained using the uniform prior time distribution of strategy 1 (Table 1). In contrast, calibration densities modeled with the Cauchy distribution (strategies 3 and 4) exhibit strong truncation effects in the time priors (Figures 1B and 1C), resulting in substantially older time estimates (Figure 1D). This can be seen, for example, in association with crown Bilateria, Deuterostomia, and Protostomia,

Table 1. Minimum and Maximum Fossil Constraints and 95% HPD Limits of Posterior Divergence Times for Various Metazoan Clades, in Millions of Years before Present

		Calibration		S1, IR, 1P		S2, IR, 1P		S1, IR, 10P		S1, AR, 1P		Composite	
Node	Crown Group	Min.	Max.	Min.	Max.	Min.	Max.	Min.	Max.	Min.	Max.	Min.	Max.
**55**	Metazoa	552.85	833	680.6	832.7	716.2	833.4	786.8	833.5	649.8	763.9	649.8	833.5
**58**	Eumetazoa	552.85	636.1	630.7	652.9	649.5	714.2	712.2	746.2	625.9	648.0	625.9	746.2
**59**	Cnidaria	529	636.1	533.3	620.5	537.7	631.9	596.2	641.7	587.4	629.0	531.5	641.8
**63**	Bilateria	552.85	636.1	615.1	637.8	624.2	672.3	665.6	688.3	595.7	618.7	595.7	688.3
**64**	Deuterostomia	515.5	636.1	593.7	627.9	598.0	649.6	639.5	662.3	587.2	610.6	587.2	662.3
**65**	Chordata	514	636.1	555.4	611.3	558.1	622.2	609.0	635.7	573.9	600.6	555.4	635.7
**66**	Olfactores	514	636.1	516.6	583.6	524.3	588.0	568.0	600.0	551.2	587.0	516.3	600.0
**68**	Vertebrata	457.5	636.1	459.6	527.9	467.1	527.6	483.3	512.9	481.4	533.8	459.3	533.8
69	Gnathostomata	420.7	468.4	432.9	468.7	433.9	468.6	436.2	451.3	440.5	468.9	432.1	468.1
70	Osteichthyes	420.7	453.7	420.6	444.1	420.6	443.9	420.6	425.0	420.6	438.1	420.6	444.2
71	Tetrapoda	337	351	338.3	351.4	338.4	351.5	346.5	352.1	345.8	352.2	338.2	354.0
72	Amniota	318	332.9	318.0	331.4	318.0	331.1	318.0	321.5	318.0	323.7	318.0	331.5
73	Mammalia	164.9	201.5	165.1	200.7	164.9	200.5	164.8	186.5	167.8	202.8	164.8	204.7
74	Euarchontoglires	61.6	164.6	61.4	140.2	61.4	135.3	61.3	67.3	61.6	124.7	61.2	140.3
75	Cyclostomata	358.5	636.1	358.1	458.0	358.1	455.8	358.3	416.5	378.1	494.3	358.0	494.3
76	Xenambulacraria	515.5	636.1	569.8	614.5	575.9	632.2	617.6	639.9	577.8	603.0	569.3	639.9
77	Ambulacraria	515.5	636.1	534.6	591.3	538.5	603.5	572.6	600.1	556.0	586.9	534.1	603.1
80	Hemichordata	504.5	636.1	504.2	537.6	504.2	540.0	504.1	511.4	504.2	535.8	504.1	540.0
**82**	Protostomia	552.85	636.1	598.0	626.4	603.6	647.5	635.3	653.5	578.1	599.0	578.1	653.1
**85**	Annelids-Molluscs	534	636.1	552.3	586.1	554.1	591.7	577.4	595.1	556.4	572.5	552.2	595.1
**86**	Capitellid-Polychete-leech	476.5	636.1	476.3	548.1	480.9	550.9	476.3	517.5	503.5	548.7	476.3	550.9
90	Mollusca	534	549	538.4	549.6	539.1	549.7	545.8	550.3	540.9	549.5	538.3	550.3
91	Bivalve-Gastropod	530	549	530.0	539.1	530.0	538.6	530.0	532.6	530.0	536.9	530.0	539.2
**92**	Gastropoda	470.2	549	470.0	508.3	470.3	506.2	470.0	478.8	470.5	512.6	470.0	512.6
96	Ecdysozoa	528.82	636.1	577.8	613.2	581.9	627.1	608.8	628.9	566.5	585.8	566.5	628.9
**97**	Nematoda-Arthropoda	528.82	636.1	561.4	599.8	563.8	608.3	589.8	610.4	557.2	575.5	557.2	610.4
98	Lobopodia	528.82	636.1	545.1	582.8	547.8	588.5	568.5	587.0	546.1	561.7	545.1	588.5
**99**	Euarthropoda	514	636.1	530.8	559.4	531.9	560.7	543.3	556.2	533.0	540.9	530.8	560.7
100	Mandibulata	514	531.22	523.4	532.3	524.0	532.3	530.3	536.1	528.1	532.8	523.4	536.1
101	Pancrustacea	514	531.22	514.0	522.8	514.0	522.3	514.0	517.5	514.0	517.6	514.0	522.8
102	Copepoda-Branchiopoda	499	531.22	499.0	510.1	498.9	509.2	498.9	500.5	499.0	506.4	498.9	510.1
105	Eumetabola	305.5	413.6	305.3	396.8	305.3	393.1	305.3	335.8	318.5	418.3	305.2	418.3
106	Pycnogonida-other chelicertates	497.5	531.22	497.5	526.1	497.5	525.8	497.4	509.1	497.5	518.5	497.4	526.3
107	Acari-Arenacea	416	531.22	415.9	479.9	415.8	477.5	415.8	436.4	419.6	492.5	415.7	492.5

Nodes are numbered as in Figure 6. Note: posterior times are the 95% highest probability density (HPD) interval, estimated with MCMCTree v4.8 under the LG + Γ_4 + F model. S1, calibration strategy 1; S2, strategy 2; IR, independent rates model; AR, autocorrelated rates model; 1P, the 203 proteins analyzed as a single partition; 10P, the proteins are grouped into ten partitions according to their evolutionary rates. Nodes in bold have calibrations that differ in strategy 1 and strategy 2. Composite: 95% confidence interval (CI) is a composite of the 95% CI across all analysis, except those under strategy 3 and strategy 4 and under alternative topologies.

where truncation caused the effective priors to place considerable probability mass beyond the maximum bound of 636.1 Ma (Figure 1C). This differs significantly from the specified calibration densities (cf. Figure 1B), resulting in posterior time estimates that are substantially older than those derived using strategies 1 and 2 (Figure 1D). For example, estimates under strategy 3 indicate Metazoa originated 834–795 Ma, Bilateria 759–685 Ma, Deuterostomia 722–644 Ma, and Protostomia 712–644 Ma (Table S3, cf. strategy 1) Thus, truncation can have dramatic and perhaps surprising effects. These effects may be hard to predict, highlighting the challenges in constructing fossil calibrations, as calibrations based on the same fossil information can unintentionally lead to dramatically different priors and posterior estimates of divergence times.

Age estimates for the younger nodes are similar under all four calibration strategies (e.g., nodes 68, 86, and 92; Table S3). However, the posterior age estimates of nodes close to the root exhibit dramatic differences among the different calibration

CellPress

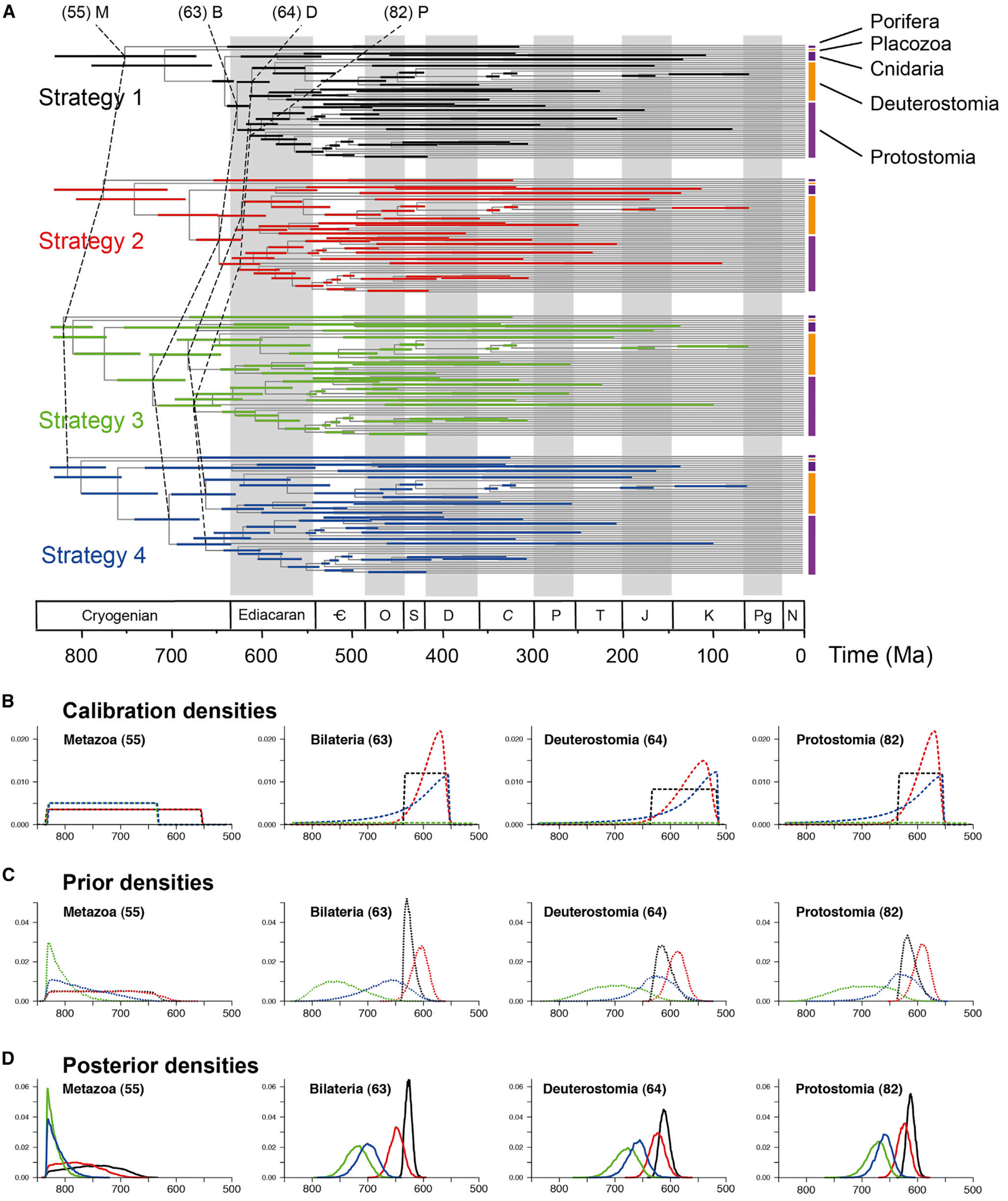

Figure 1. The Effect of Fossil Calibrations on Posterior Divergence Time Estimates of Metazoans

(A) Time trees showing posterior divergence time estimates for major metazoan groups. Nodes are drawn at the posterior means obtained and horizontal bars represent 95% HPD intervals. Estimates were obtained with MCMCTree using the LG + Γ_4 + F model, IR, and with the 203 proteins concatenated into a super alignment.
(B–D) Calibration, prior, and posterior densities for four ancient nodes in the metazoan phylogeny; coloring relates to the calibration strategy employed as in (A).
(The phylogeny with species names is provided in Figure 6.)

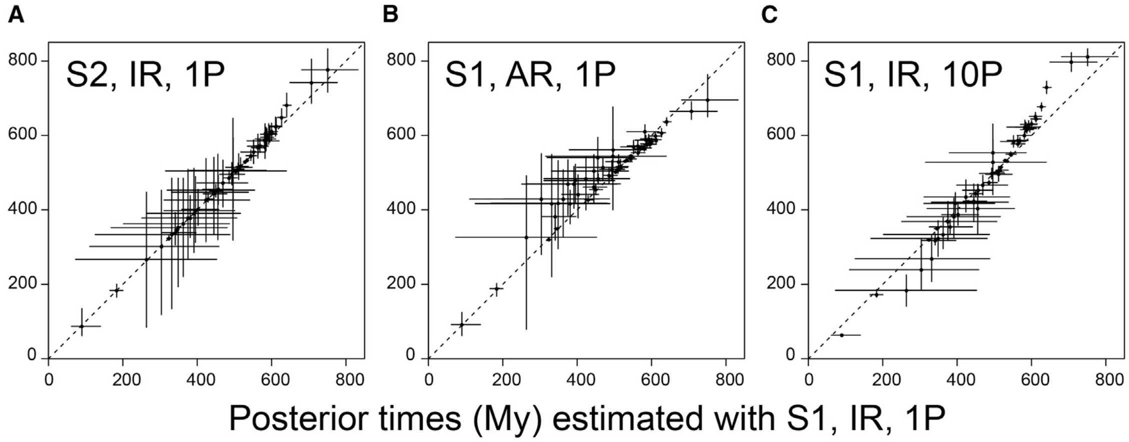

Figure 2. Sensitivity of Time Estimates to Fossil Calibrations, Rate Model, and Number of Partitions

The posterior mean times estimated under calibration strategy 1, independent rates (IR) model, and a single partition are plotted against (A) estimates using strategy 2, (B) estimates under the autocorrelated rates (AR) model, and (C) estimates obtained when the 203 gene alignments are divided into ten partitions according to substitution rate. The bars indicate the 95% HPDs.

strategies (e.g., Figure 2A). This appears to reflect a paucity of palaeontological evidence, requiring very different scenarios for the timing and tempo of metazoan diversification. Strategies 3 and 4 yield timescales that strongly favor an early Cryogenian (834–780 Ma) diversification, evidently constrained by the root age, while the age estimates arising from calibration strategies 1 and 2 are compatible with metazoans diversifying at any time within the Cryogenian, though these analyses are not otherwise very informative (Figure 1D).

Calibration strategies 1–4 are based on a protostome interpretation of the Ediacaran *Kimberella* (552.85 Ma), to constrain the minimum time of divergence of Protostomia, Bilateria, Eumetazoa, and Metazoa (Table 1). However, to some, there is no unequivocal fossil evidence of metazoans prior to the Cambrian. In this view, interpreting *Kimberella* as a protostome leads to unduly ancient estimates for the origin of all the more universal clades encompassing Protostomia. To assess the impact of using *Kimberella* as a minimum constraint on the age of the protostome clade, we employed a variation of calibration strategy 1 in which the next-oldest record of Protostomia and oldest unequivocal total-group mollusc, the Cambrian *Aldanella yanjiahensis* (532 Ma), was used in place of *Kimberella*. The resulting divergence time estimates are effectively the same as those derived using strategy 1 (Figure S3). Thus, even under the assumption that the fossil record of metazoan is limited to the Cambrian, our estimates require an Ediacaran origin for most crown-bilaterian phyla, a late Cryogenian-early Ediaracan origin of crown Bilateria, and an early Cryogenian origin of crown Metazoa.

The Impact of Strong Violations of the Molecular Clock in Ancient Timescales

When rate variation across a phylogeny is extreme (that is, when the molecular clock is seriously violated), the rates calculated on one part of the phylogeny will serve as a poor proxy for estimating divergence times in other parts of the tree. In such instances, divergence time estimation is challenging and the analysis becomes sensitive to the rate model used.

To examine the impact of this uncertainty, we re-estimated the divergence times of metazoans assuming an autocorrelated rates (AR) model [53] under calibration strategy 1. This relaxed-clock model imposes a correlation of rates between ancestral and descendant branches by modeling rate change on the tree as a geometric Brownian diffusion process [53, 54]. We found that the choice between AR versus IR relaxed-clock models has a strong impact on the estimated divergences (Table 1 and Figure 2B). Our results show that many posterior time estimates for young nodes using the AR model are older than those derived using the IR model, whereas a few nodes, especially the deep nodes, are younger (Table 1 and Figure 2B). In particular, the divergences of crown Metazoa (764–650 Ma), crown Bilateria (619–596 Ma), crown Deuterostomia (611–587 Ma) and crown Protostomia (599–578 Ma) are substantially younger.

The AR model penalizes extreme rate variation over short time intervals and effectively imposes local clocks for closely related species while allowing large rate variation among distant clades. This contrasts with the IR model, which assumes that the variance of the rate is independent of the divergence time, so that the variance is the same whether the species are closely or distantly related. Figure 3 shows the change in the shape of the log-normal distribution of rates under the AR model across 500 million years of evolution and highlights the extreme level of rate variation in Metazoan phylogeny. At short timescales, the distribution is more symmetrical and has a smaller variance than at longer timescales. In the case of the IR model with μ = 0.089/100 million years and σ^2 = 0.468/100 million years, the log-normal distribution has the same shape as that for 100 million years for the AR (Figure 3, third plot).

Which clock model should be used? Bayes factors have been used to decide between competing clock models (such as the IR and AR models, e.g., [55]) in phylogenetic analysis. MCMCTree does not yet implement Bayes factors, and so we did not calculate them here. Further work will be required to assess the suitability of the various clock models to describe rate evolution in the metazoan phylogeny. Thus, in attempting to encompass

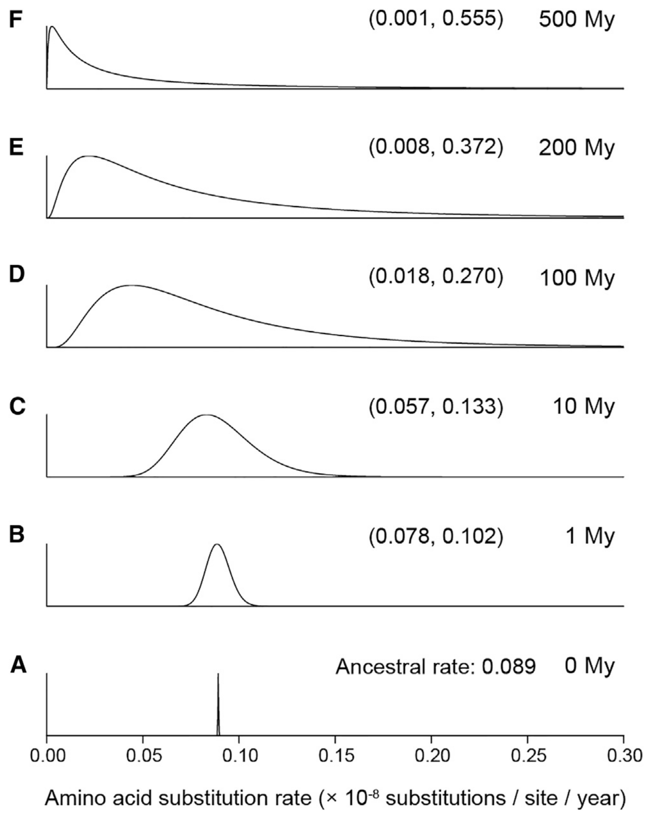

Figure 3. Explosive Relaxation of Molecular Rates during Metazoan Evolution

In the AR model, the rates at the tips of a star phylogeny are log-normally distributed with mean r_A (the ancestral rate at the root) and log-variance of the rate $\sigma^2 = t\nu$. For the metazoan phylogeny, the posterior mean of r_A is 0.089 s/s/100 million years and of v is 0.468/100 million years. In (A)–(F), the evolution of the rate of molecular evolution is shown through 500 million years of metazoan history assuming the AR model to be correct. The numbers in brackets are the 95% equal-tail range of the distribution of the rate for the given time. As the star phylogeny evolves, the variance of the rates increases exponentially. After 500 million years of evolution, the 95% equal-tail range encompasses two orders of magnitude. Note that in case of the IR model with $\mu = 0.089/100$ million years and $\sigma^2 = 0.468/100$ million years, the shape of the log-normal distribution is the same as that for 100 million years for the AR at any time point. Note that here the timescale is given in million years from the root (i.e., 0 million years is the root, and 500 million years is present time).

the uncertainty in the rate drift model, we consider here the spread of node age estimates that arise from both rate models.

The Impact of Data Partitioning

Partitioning of the molecular sequence alignment may impact on divergence time estimates [56, 57]. To explore this, the protein alignment was divided into two, four, five, and ten partitions, according to the relative amino acid substitution rates among genes (see the Supplemental Experimental Procedures). The posterior mean times for the most ancient nodes tended to increase as the number of partitions increases (Figure 2C). For example, divergence time estimates for crown Metazoa vary from 833–681 Ma (single partition) to 834–787 Ma (ten partitions; Table 1). The discrepancy between age estimates increases with proximity to the root, regardless of whether or not the nodes are calibrated (Figure 2C). Age estimates on intermediate nodes (e.g., all vertebrates and most arthropod nodes) do not vary significantly with partition strategy; for a small number of nodes, younger date estimates were obtained when more partitions were used (Figure 2C and Table 1). Overall, nodes with highly variable time estimates among different partitions are those without calibration or are close to the root, where the calibrations are least informative (Table S4 and Figure S2).

Figure 4 shows the so-called infinite-sites plot in which the width of the 95% HPD interval is plotted against the posterior mean. The precision of node age estimates, as reflected in the 95% HPD interval, increases with the number of partitions (Figure 4). Dividing the data into more partitions gives narrow HPD intervals, as indicated by the reduced regression coefficients in the plot. The extent of this reduction diminishes with higher numbers of partitions (for example, compare four, five, and ten partitions), indicating that, given the fixed set of calibrations and fixed sequence data, the number of partitions may already be near optimal in terms of dating precision. Nodes with the widest HPD interval are those with no fossil calibrations, indicating that including more calibration points is likely to improve the precision of the time estimates. Finally, since the plots are very scattered (very low R^2 values), adding more sequence data may lead to smaller HPDs, and hence more precise node age estimates.

Impact of Phylogenetic Uncertainty

All of the preceding analyses employed a fixed tree topology (Figure 1), yet the phylogenetic position of some metazoan taxa remains the subject of debate [58]. To account for this uncertainty, we analyzed 161 alternative binary trees, accounting for uncertainties in the positioning of Bilateria, chaetognaths, molluscs, nematodes, and xenacoelomorphs. The results of these analyses show that nodes are affected differently depending on the tree topology. For example, some nodes are characterized by time estimates that remain similar across all topologies (Figure 5). These nodes are usually well calibrated and/or the local phylogeny well accepted, such as in crown deuterostomes and arthropods (Figure 5). In contrast, nodes with uncertain phylogenetic relationships exhibit considerable variation in estimated ages. These include the nodes close to the root of the tree, such as Metazoa, Bilateria, and Cnidaria; this variation increases with proximity to the root. For example, moving the position of Placozoa around the eumetazoan node has a profound impact on the estimated age of the root (Figure 5).

DISCUSSION

The timing of the emergence of animals has troubled evolutionary biologists at least since Darwin, who was sufficiently incredulous that he considered the abrupt appearance of animal fossils in the Cambrian as a challenge to his theory of evolution by natural selection [59]. There has been, as a result, a long history of attempts to rationalize a rapid radiation of animals through theories of non-uniform evolutionary processes, such as homeotic mutations, removal of environmental restrictions on larger body sizes, through to the assembly of gene regulation kernels—proposed both as an explanation for rapid rates of innovation followed by subsequent constraint against fundamental innovation of new body plans after the Cambrian [60, 61]. Indeed,

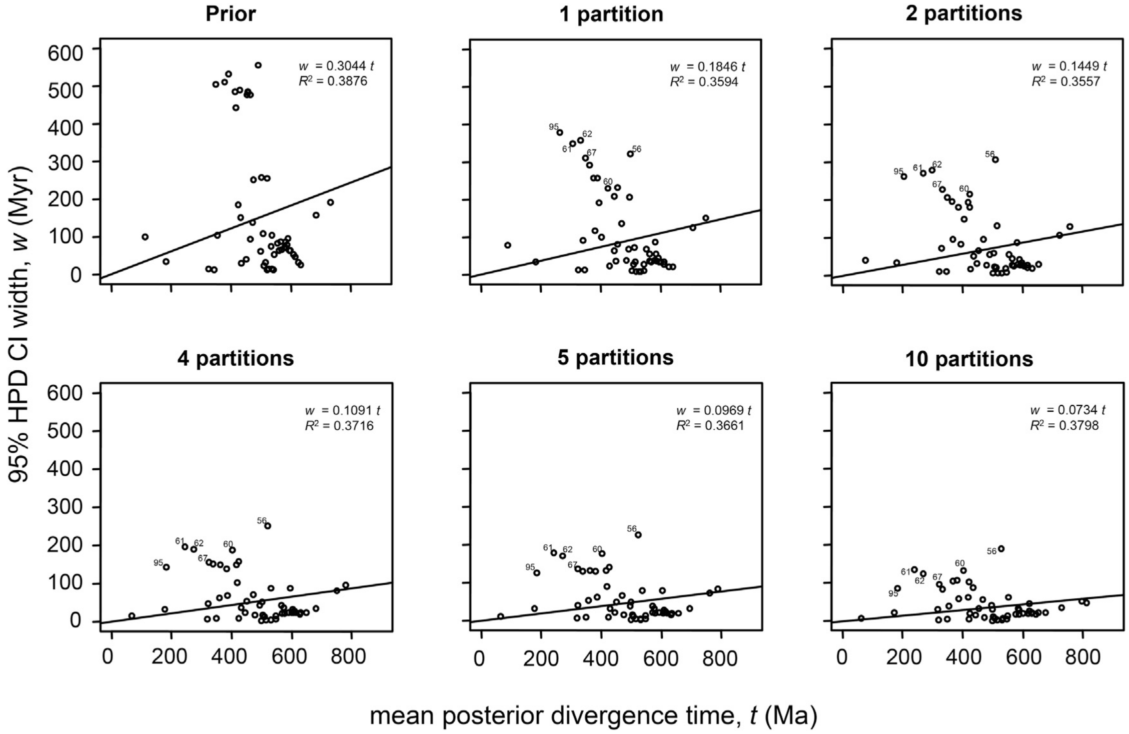

Figure 4. Infinite-Sites Plots

The 95% HPD width is plotted against the mean of the divergence times estimated without molecular data (prior) and with the 203 gene alignments divided into one, two, four, five, and ten partitions. This plot indicates how much of the uncertainty in the posterior time estimates is due to the uncertain fossil calibrations and how much is due to the limited amount of sequence data. Thus, the low correlations indicate that the limited amount of sequence data contributes substantially to posterior uncertainty and the regression coefficients also indicate that the fossil calibrations involve much uncertainty.

there have been explicit attempts to accommodate rapid rates of phenotypic evolution in the early Cambrian, compatible with these hypotheses and a semi-literal (albeit phylogenetically constrained) reading of the fossil record [38].

And yet our results, as have others before them, suggest that there is no justification for invoking non-uniform mechanisms to explain the emergence of animals and their phylum-level body plans. Our analysis attempts to integrate different interpretations of the animal fossil record in informing the minimum age of animal clades. Some of these identify fossil evidence of animals extending into the Cryogenian [20, 62], whereas, at the other extreme, others argue that coherent evidence of animals is limited to the Cambrian or the terminal few millions of years of the Neoproterozoic [63]. Although a case may be made for the restriction of animal fossils to the Phanerozoic, there is only negative evidence (an absence of uncontroversial animal fossils) supporting a Cambrian explosion of animals. This is the long-standing conundrum of the Cambrian—whether the first animal fossils faithfully reflect an explosion in animal biodiversity or merely an explosion of fossils [64]. The results of our study—which integrates fossil and molecular evidence to establish an evolutionary timescale—suggest that the Cambrian explosion is a phenomenon of fossilization, while biological diversity was established in the Neoproterozoic. Integrating all of the sources of uncertainty that we explore (Figure 6, Table 1) allows us to conclude that crown Metazoa originated 833–650 Ma, fully within the Cryogenian, while the component clades of crown Eumetazoa (746–626 Ma), crown Bilateria (688–596) Ma, crown Deuterostomia (662–587 Ma), and crown Protostomia (653–578 Ma) all diverged within a Cryogenian to early- or mid-Ediacaran interval.

The results of our analyses leads us to reject the hypothesis that metazoans, eumetazoans, bilaterians, protostomes, deuterostomes, ecdysozoans, lophotrochozoans, or, for that matter, any of the component phylum-level total groups, originated in the Cambrian. The uncertainties from competing interpretations of the fossil record, through the choice of rate models and sequence partition strategies, to competing phylogenetic hypotheses all contribute to an evolutionary timescale that lacks sufficient precision to rule out many hypotheses. The situation is compounded by at least two additional sources of uncertainty that we did not study here: uncertainty introduced by the birth-death time prior, and its failure to accommodate diversified sampling of species in phylogenies [65], and uncertainty due to the substitution model, which may have an important effect when estimating branch lengths in ancient phylogenies [66, 67]. Some of the uncertainty in metazoan divergence times can be reduced, for example, by the addition of more sequence data, constraining local rate variation rate through the addition of more taxa. However, the improvements in precision possible even with genome-scale sequence data will be limited by the confounding effects of time and rate, which is the crux of the problem.

No matter how imprecise, our timescale for metazoan diversification still indicates a mismatch between the fossil evidence

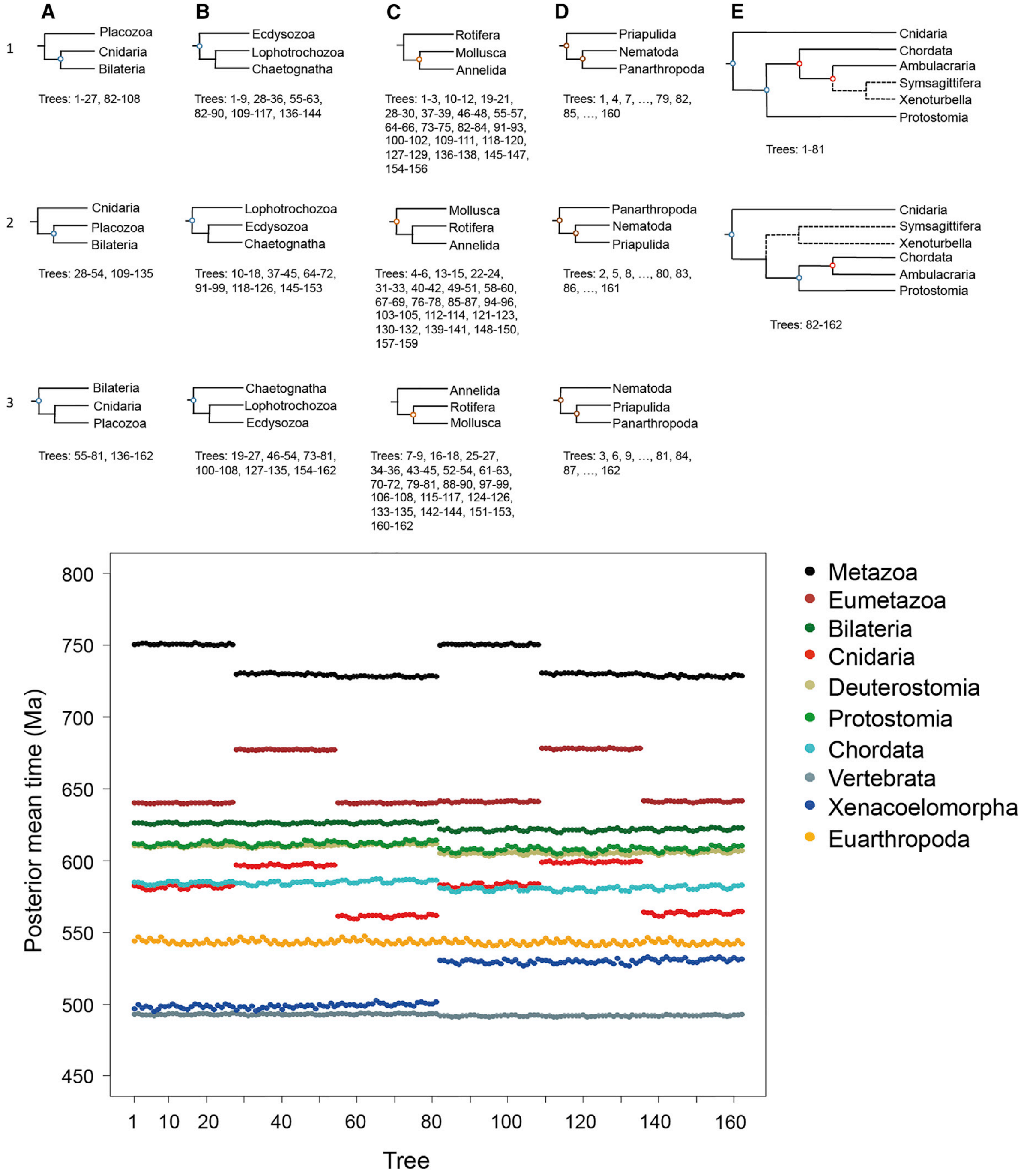

Figure 5. Effect of Uncertainty in Tree Topology on Divergence Time Estimates of the Metazoa

Four nodes (A–D) can be rearranged in three different ways (1–3), and a fifth node (E) can be rearranged in two ways, resulting in a total of 162 tree topologies reflecting the uncertain relationships around these five nodes. Divergence times were estimated using strategy 1, the IR model, and a single partition using each tree (bottom panel). Some phylogenetic hypothesis had a strong effect on posterior mean times; for example, placing the Placozoa as the most basal with respect to Cnidaria and Bilateria (A) leads to substantially older divergence times for the Metazoa (bottom panel), whereas placing Cnidaria as the most basal leads to substantially older times for the divergence of Eumetazoa.

used to calibrate the molecular clock analyses and the resulting divergence time estimates. This is not altogether surprising since, by definition, minimum constraints of clade ages anticipate their antiquity. Nevertheless, it is the extent of this prehistory that is surprising, particularly since the conditions required for exceptional fossil preservation, so key to evidencing the existence of animal phyla in the early Cambrian, obtained also in the Ediacaran [68]. However, the early Cambrian is characterized

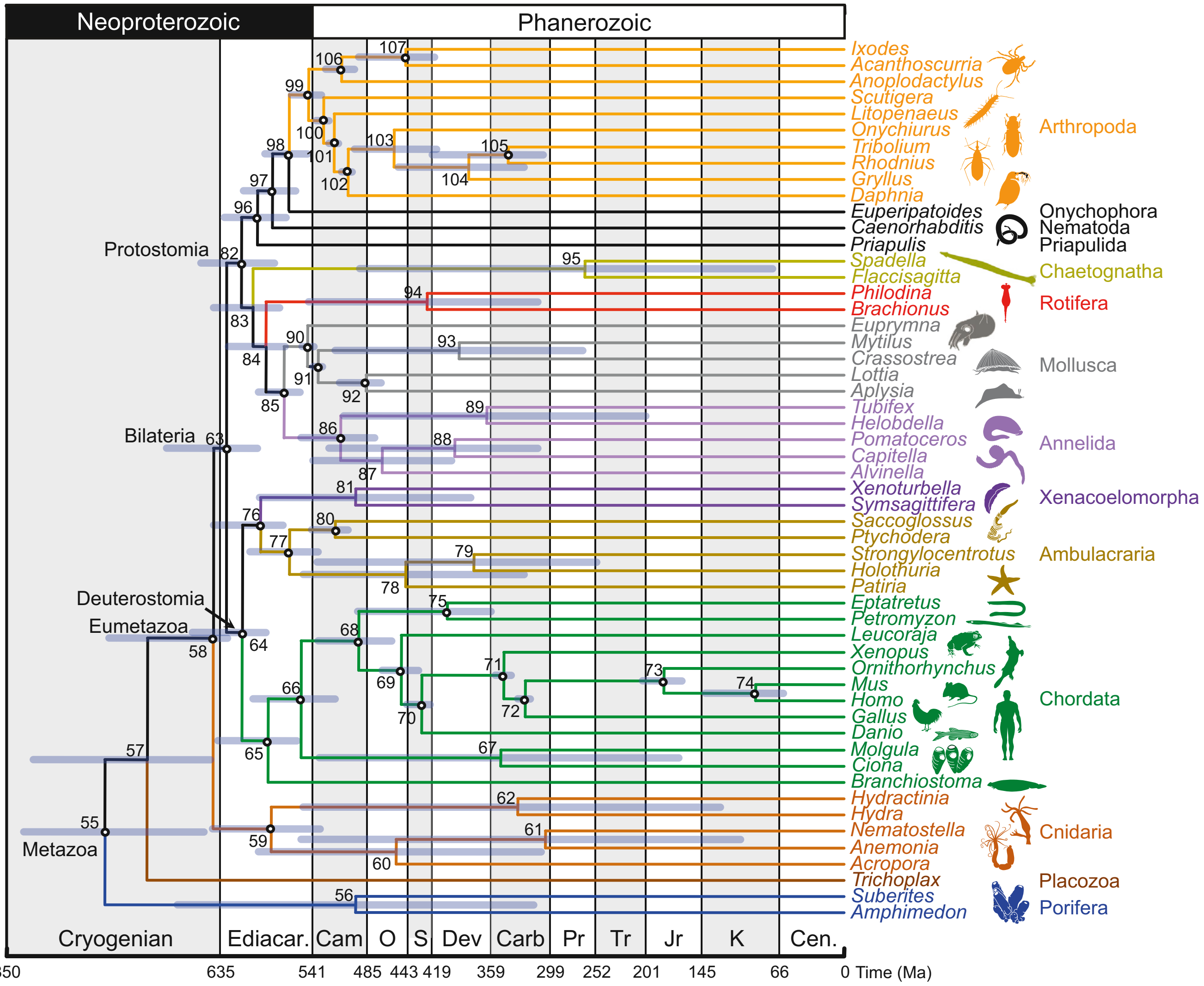

Figure 6. The Timetree of the Metazoa Encompassing Major Sources of Uncertainty in Time Estimates

Node ages are plotted at the posterior mean for the calibration strategy 1, one partition, IR, and LG + Γ analysis. The node bars are composites extending from the minimum 2.5% HPD limit to the maximum 97.5% limit across all analyses (excluding results from calibration strategies 3 and 4 and from alternative topologies). Cen, Cenozoic; K, Cretaceous; Jr, Jurassic; Tr, Triassic; Pr, Permian; Carb, Carboniferous; Dev, Devonian; S, Silurian; O, Ordovician; Cam, Cambrian; Ediacar, Ediacaran.

by a global sea level rise associated with increased tectonic activity leading to the destruction of older rock sequences by erosion and subduction. Although this may have promoted the innovation and radiation of skeletonizing animals [14], it will also have diminished the fossil record of their forebears [9]. That said, there remains a record of metazoan- and bilaterian-like fossil remains and traces in the Ediacaran that we considered insufficiently robust to substantiate a minimum constraint on metazoan clades but that invariably informed maxima. Further insights into the biology of these organisms and others like them may well explain away apparent inconsistencies between molecular clock estimates of deep metazoan clade ages and their fossil record.

Nevertheless, attempts to build evolutionary narratives of animal evolution based on recent molecular clock studies appear to be premature. They fail to integrate different sources of uncertainties, which make accurate and precise divergence time estimates impossible with current data and methods. Progress may be possible through analysis of combined morphological and molecular data, which allow fossil species to be integrated into divergence time analyses on par with their living relatives [69, 70]. Combined analyses are expected to reduce uncertainty in prior node ages as compared to traditional analysis based on simplistic fossil-based constraints [71, 72]. However, most such analyses conducted to date have yielded unacceptably old divergence time estimates, even older than traditional node-calibrated studies [73]. Otherwise, statistical analyses of fossil stratigraphic data may yield more objective time priors (e.g., [74–76]) and more informative calibrations. Above all, establishing unequivocal evidence for

the presence of metazoan clades in the late Neoproterozoic, as well as for the absence in more ancient strata, will probably have more impact than any methodological advance in improving the accuracy and precision of divergence time estimates for deep metazoan phylogeny. Realizing the aim of a timescale of early animal evolution that is not merely accurate, but sufficiently precise to effect tests of hypotheses on the causes and consequences of early animal evolution, will require improved models of trait evolution and improved algorithms to allow analysis of genome-scale sequence data in tandem with morphological characters.

EXPERIMENTAL PROCEDURES

Molecular Data Assembly

Two independent molecular datasets [35, 47] were combined into a single amino acid alignment. The alignments were updated with additional proteins from GenBank to include five additional species (*Homo sapiens*, *Mus musculus*, *Ornithorhynchus anatinus*, *Tribolium castaneum*, and *Caenorhabditis elegans*). Sequences were re-aligned [77], and alignment gaps were removed [78]. The combined alignment consists of 203 nuclear encoded proteins (38,577 amino acid positions) from 71 species (missing data 21.49%). This process recovered the original alignments but included extra species and sequences of genes previously missing or incomplete. The alignment was also divided into two, four, five, and ten partitions according to the relative evolutionary rates of proteins (measured by the distance between *Hydra magnipapillata* and *Strongylocentrotus purpuratus*).

Tree Topology

As the relationships among many taxa remain unresolved, 17 species were removed from the dataset to reduce the uncertainty in the topology. This resulted in a smaller alignment of the remaining 54 species (missing data 13.97%). The phylogeny for these 54 species has four uncertain nodes that can be rearranged in three ways and one uncertain node that can be rearranged in two ways, giving $3^4 \times 2 = 162$ possible fully resolved trees that were used for analysis. One of those trees (Figure 1A), mainly based on [32] with adjustments based on more recent discoveries and known controversies, was chosen for the main analysis, whereas the other 161 trees were used to assess the robustness of the time estimates to the various topologies.

Fossil Calibrations

Thirty-four minimum and maximum fossil age constraints were derived from [79] with updates [49, 80]. The minimum ages were determined from the oldest uncontroversial record belonging to one of the two sister clades. These inferred minima are conservative, and the actual origination time of a clade is likely to be older. The maximum ages were derived from the base of the youngest stratigraphic range or geological formation known not to contain any members of the clade of interest [81, 82]. On the basis of these maximum and minimum bounds, we constructed calibration densities for four calibration strategies. In strategy 1, the 34 calibrations are represented as uniform distributions with soft bounds [50]. In strategy 2, 13 calibrations are represented by skew-normal distributions, with the minimum and maximum bounds matching the corresponding quantiles of the distribution; the rest of the calibrations are as in strategy 1. Strategy 3 is like strategy 2, but the truncated Cauchy distribution with a long tail [51] is used instead of the skew normal. Strategy 4 is like strategy 3, but the tail of the truncated Cauchy is shorter. The detailed strategies are presented in Table S1.

Divergence Time Estimation

Molecular dating was performed using the program MCMCTree v4.8 [52]. The time unit used was 100 million years. The prior on times was constructed using the fossil calibrations combined with the birth-death process [50] with parameters $\lambda = \mu = 1$, $\rho = 0$ (representing a uniform distribution of node ages given the root age).

Because the molecular alignment is large, the likelihood was calculated approximately to save computing time [54, 83]. The approximation uses the gradient and Hessian matrix of the likelihood at the maximum likelihood estimates of branch lengths. These were calculated with the program CODEML [52] using the LG + Γ_4 + F amino acid substitution model [84, 85].

Both the IR and the AR models were used [53]. The prior on the mean rate (or the ancestral rate) was set to G(2, 40). This is a diffuse prior with the mean to be 0.05 (or 5×10^{-10} amino acid substitutions per site per year). The overall mean was derived from the average pairwise amino acid distances between the 203 proteins of *Hydra magnipapillata* and *Strongylocentrotus purpuratus* assuming a divergence time around 636.1 Ma. The prior for σ^2 was set to G(1, 10), indicating serious violation of the clock. The priors were set using the gamma-Dirichlet prior [56].

The number of iterations, the burn-in, and the sampling frequency were adjusted in test runs of the program. In addition, at least two chains were run to ensure convergence. Convergence was assessed by comparing the posterior means and plotting the time series traces of the samples from two independent runs. The resulting posterior distribution was summarized as the means and 95% HPD intervals.

Detailed methods are given in the Supplemental Experimental Procedures.

ACCESSION NUMBERS

The molecular sequence alignment and trees with fossil calibrations have been deposited in Figshare: http://dx.doi.org/10.6084/m9.figshare.1525089.

SUPPLEMENTAL INFORMATION

Supplemental Information includes Supplemental Experimental Procedures, three figures, and four tables and can be found with this article online at http://dx.doi.org/10.1016/j.cub.2015.09.066.

AUTHOR CONTRIBUTIONS

M.d.R, M.J.T., P.C.J.D., and Z.Y. conceived the project and designed the analysis. Y.T. prepared the data and set up the analysis pipeline. Y.T., K.A., and M.d.R. analyzed the data. P.C.J.D., Y.T., and M.d.R. wrote the main draft of the manuscript. All authors contributed to the interpretation of results and worked on the manuscript.

ACKNOWLEDGMENTS

We thank Alex Liu and Davide Pisani (Bristol Palaeobiology) for providing useful feedback on a draft manuscript. This work was supported by the Biotechnology and Biological Sciences Research Council (UK) grant BB/J009709/1. Y.T. was supported by a Royal Thai Government scholarship. K.A. was supported by a UCL Impact studentship. M.J.T. and P.C.J.D. are supported by Royal Society Wolfson Research Merit Awards.

Received: June 9, 2015
Revised: September 20, 2015
Accepted: September 22, 2015
Published: October 22, 2015

REFERENCES

1. Signor, P.W., and Lipps, J.H. (1992). Origin and early radiation of the Metazoa. In Origin and Early Evolution of the Metazoa, P.W. Signor, and J.H. Lipps, eds. (Springer), pp. 3–23.
2. Sebé-Pedrós, A., de Mendoza, A., Lang, B.F., Degnan, B.M., and Ruiz-Trillo, I. (2011). Unexpected repertoire of metazoan transcription factors in the unicellular holozoan Capsaspora owczarzaki. Mol. Biol. Evol. *28*, 1241–1254.
3. Planavsky, N.J., Reinhard, C.T., Wang, X., Thomson, D., McGoldrick, P., Rainbird, R.H., Johnson, T., Fischer, W.W., and Lyons, T.W. (2014). Earth history. Low mid-Proterozoic atmospheric oxygen levels and the delayed rise of animals. Science *346*, 635–638.
4. Knoll, A.H., and Carroll, S.B. (1999). Early animal evolution: emerging views from comparative biology and geology. Science *284*, 2129–2137.

5. Hoffman, P.F., and Schrag, D.P. (2002). The snowball Earth hypothesis: testing the limits of global change. Terra Nova *14*, 129–155.

6. Shen, Y., Zhang, T., and Hoffman, P.F. (2008). On the coevolution of Ediacaran oceans and animals. Proc. Natl. Acad. Sci. USA *105*, 7376–7381.

7. Kataoka, R., Ebisuzaki, T., Miyahara, H., Nimura, T., Tomida, T., Sato, T., and Maruyama, S. (2014). The Nebula Winter: the united view of the snowball Earth, mass extinctions, and explosive evolution in the late Neoproterozoic and Cambrian periods. Gondwana Res. *25*, 1153–1163.

8. Kirschvink, J.L., Ripperdan, R.L., and Evans, D.A. (1997). Evidence for a large-scale reorganization of Early Cambrian continental masses by inertial interchange true polar wander. Science *277*, 541–545.

9. Dalziel, I.W.D. (2014). Cambrian transgression and radiation linked to an Iapetus-Pacific oceanic connection? Geology *42*, 979–982.

10. Li, C., Love, G.D., Lyons, T.W., Fike, D.A., Sessions, A.L., and Chu, X. (2010). A stratified redox model for the Ediacaran ocean. Science *328*, 80–83.

11. Knauth, L.P. (1998). Salinity history of the Earth's early ocean [letter]. Nature *395*, 554–555.

12. Knauth, L.P. (2005). Temperature and salinity history of the Precambrian ocean: implications for the course of microbial evolution. Palaeogeogr. Palaeoclimatol. Palaeoecol. *219*, 53–69.

13. Anbar, A.D., and Knoll, A.H. (2002). Proterozoic ocean chemistry and evolution: a bioinorganic bridge? Science *297*, 1137–1142.

14. Peters, S.E., and Gaines, R.R. (2012). Formation of the 'Great Unconformity' as a trigger for the Cambrian explosion. Nature *484*, 363–366.

15. McKenzie, N.R., Hughes, N.C., Gill, B.C., and Myrow, P.M. (2014). Plate tectonic influences on Neoproterozoic-early Paleozoic climate and animal evolution. Geology *42*, 127–130.

16. Peterson, K.J., and Butterfield, N.J. (2005). Origin of the Eumetazoa: testing ecological predictions of molecular clocks against the Proterozoic fossil record. Proc. Natl. Acad. Sci. USA *102*, 9547–9552.

17. Sperling, E.A., Frieder, C.A., Raman, A.V., Girguis, P.R., Levin, L.A., and Knoll, A.H. (2013). Oxygen, ecology, and the Cambrian radiation of animals. Proc. Natl. Acad. Sci. USA *110*, 13446–13451.

18. Mills, D.B., and Canfield, D.E. (2014). Oxygen and animal evolution: did a rise of atmospheric oxygen "trigger" the origin of animals? BioEssays *36*, 1145–1155.

19. Lenton, T.M., Boyle, R.A., Poulton, S.W., Shields-Zhou, G.A., and Butterfield, N.J. (2014). Co-evolution of eukaryotes and ocean oxygenation in the Neoproterozoic era. Nat. Geosci. *7*, 257–265.

20. Love, G.D., Grosjean, E., Stalvies, C., Fike, D.A., Grotzinger, J.P., Bradley, A.S., Kelly, A.E., Bhatia, M., Meredith, W., Snape, C.E., et al. (2009). Fossil steroids record the appearance of Demospongiae during the Cryogenian period. Nature *457*, 718–721.

21. Siegl, A., Kamke, J., Hochmuth, T., Piel, J., Richter, M., Liang, C., Dandekar, T., and Hentschel, U. (2011). Single-cell genomics reveals the lifestyle of Poribacteria, a candidate phylum symbiotically associated with marine sponges. ISME J. *5*, 61–70.

22. Xiao, S., Zhang, Y., and Knoll, A.H. (1998). Three-dimensional preservation of algae and animal embryos in a Neoproterozoic phosphorite. Nature *391*, 553–558.

23. Butterfield, N.J. (2011). Paleontology. Terminal developments in Ediacaran embryology. Science *334*, 1655–1656.

24. Huldtgren, T., Cunningham, J.A., Yin, C., Stampanoni, M., Marone, F., Donoghue, P.C.J., and Bengtson, S. (2011). Fossilized nuclei and germination structures identify Ediacaran "animal embryos" as encysting protists. Science *334*, 1696–1699.

25. Zhang, X.-G., and Pratt, B.R. (2014). Possible algal origin and life cycle of Ediacaran Doushantuo microfossils with dextral spiral structure. J. Paleontol. *88*, 92–98.

26. Yin, Z., Zhu, M., Davidson, E.H., Bottjer, D.J., Zhao, F., and Tafforeau, P. (2015). Sponge grade body fossil with cellular resolution dating 60 Myr before the Cambrian. Proc. Natl. Acad. Sci. USA *112*, E1453–E1460.

27. Antcliffe, J.B., Callow, R.H., and Brasier, M.D. (2014). Giving the early fossil record of sponges a squeeze. Biol. Rev. Camb. Philos. Soc. *89*, 972–1004.

28. Fedonkin, M.A., Simonetta, A., and Ivantsov, A.Y. (2007). New data on Kimberella, the Vendian mollusc-like organism (White Sea region, Russia): palaeoecological and evolutionary implications. Geol. Soc. Lond. Spec. Publ. *286*, 157–179.

29. Liu, A.G., Matthews, J.J., Menon, L.R., McIlroy, D., and Brasier, M.D. (2014). Haootia quadriformis n. gen., n. sp., interpreted as a muscular cnidarian impression from the Late Ediacaran period (approx. 560 Ma). Proc. Biol. Sci. *281*, 20141202.

30. Carbone, C., and Narbonne, G.M. (2014). When life got smart: the evolution of behavioral complexity through the Ediacaran and Early Cambrian of NW Canada. J. Paleontol. *88*, 309–330.

31. Mángano, M.G., and Buatois, L.A. (2014). Decoupling of body-plan diversification and ecological structuring during the Ediacaran-Cambrian transition: evolutionary and geobiological feedbacks. Proc. Biol. Sci. *281*, 20140038.

32. Liu, A.G., McIlroy, D., and Brasier, M.D. (2010). First evidence for locomotion in the Ediacara biota from the 565 Ma Mistaken Point Formation, Newfoundland. Geology *38*, 123–126.

33. Rogov, V.I., Marusin, V., Bykova, N., Goy, Y., Nagovitsin, K.E., Kochnev, B.B., Karlova, G.A., and Grazhdankin, D. (2012). The oldest evidence of bioturbation on Earth. Geology *40*, 395–398.

34. Pecoits, E., Konhauser, K.O., Aubet, N.R., Heaman, L.M., Veroslavsky, G., Stern, R.A., and Gingras, M.K. (2012). Bilaterian burrows and grazing behavior at >585 million years ago. Science *336*, 1693–1696.

35. Erwin, D.H., Laflamme, M., Tweedt, S.M., Sperling, E.A., Pisani, D., and Peterson, K.J. (2011). The Cambrian conundrum: early divergence and later ecological success in the early history of animals. Science *334*, 1091–1097.

36. Budd, G.E. (2008). The earliest fossil record of the animals and its significance. Philos. Trans. R. Soc. Lond. B Biol. Sci. *363*, 1425–1434.

37. Maloof, A.C., Porter, S.M., Moore, J.L., Dudas, F.O., Bowring, S.A., Higgins, J.A., Fike, D.A., and Eddy, M.P. (2010). The earliest Cambrian record of animals and ocean geochemical change. Geol. Soc. Am. Bull. *122*, 1731–1774.

38. Lee, M.S., Soubrier, J., and Edgecombe, G.D. (2013). Rates of phenotypic and genomic evolution during the Cambrian explosion. Curr. Biol. *23*, 1889–1895.

39. Gould, S.J. (2000). Wonderful Life: The Burguess Shale and the Nature of History (Vintage).

40. Runnegar, B. (1982). A molecular-clock date for the origin of the animal phyla. Lethaia *15*, 199–205.

41. Hedges, S.B., Blair, J.E., Venturi, M.L., and Shoe, J.L. (2004). A molecular timescale of eukaryote evolution and the rise of complex multicellular life. BMC Evol. Biol. *4*, 2.

42. Peterson, K.J., Lyons, J.B., Nowak, K.S., Takacs, C.M., Wargo, M.J., and McPeek, M.A. (2004). Estimating metazoan divergence times with a molecular clock. Proc. Natl. Acad. Sci. USA *101*, 6536–6541.

43. Smith, M.P., and Harper, D.A.T. (2013). Earth science. Causes of the Cambrian explosion. Science *341*, 1355–1356.

44. Erwin, D.H., and Valentine, J.W. (2013). The Cambrian Explosion: The Construction of Animal Biodiversity (Roberts & Company).

45. Hug, L.A., and Roger, A.J. (2007). The impact of fossils and taxon sampling on ancient molecular dating analyses. Mol. Biol. Evol. *24*, 1889–1897.

46. Sharpe, S.C., Eme, L., Brown, M.W., and Roger, A.J. (2015). Timing the Origins of multicellular eukaryotes through phylogenomics and relaxed molecular clock analyses. In Evolutionary Transitions to Multicellular Life, I.R. Trillo, and A.M. Nedelcu, eds. (Springer Netherlands), pp. 3–29.

47. Philippe, H., Brinkmann, H., Copley, R.R., Moroz, L.L., Nakano, H., Poustka, A.J., Wallberg, A., Peterson, K.J., and Telford, M.J. (2011). Acoelomorph flatworms are deuterostomes related to Xenoturbella. Nature *470*, 255–258.

48. Donoghue, P.C.J., and Purnell, M.A. (2009). Distinguishing heat from light in debate over controversial fossils. BioEssays *31*, 178–189.

49. Warnock, R.C., Yang, Z., and Donoghue, P.C. (2012). Exploring uncertainty in the calibration of the molecular clock. Biol. Lett. *8*, 156–159.

50. Yang, Z., and Rannala, B. (2006). Bayesian estimation of species divergence times under a molecular clock using multiple fossil calibrations with soft bounds. Mol. Biol. Evol. *23*, 212–226.

51. Inoue, J., Donoghue, P.C.H., and Yang, Z. (2010). The impact of the representation of fossil calibrations on Bayesian estimation of species divergence times. Syst. Biol. *59*, 74–89.

52. Yang, Z. (2007). PAML 4: phylogenetic analysis by maximum likelihood. Mol. Biol. Evol. *24*, 1586–1591.

53. Rannala, B., and Yang, Z. (2007). Inferring speciation times under an episodic molecular clock. Syst. Biol. *56*, 453–466.

54. Thorne, J.L., Kishino, H., and Painter, I.S. (1998). Estimating the rate of evolution of the rate of molecular evolution. Mol. Biol. Evol. *15*, 1647–1657.

55. Lepage, T., Bryant, D., Philippe, H., and Lartillot, N. (2007). A general comparison of relaxed molecular clock models. Mol. Biol. Evol. *24*, 2669–2680.

56. Dos Reis, M., Zhu, T., and Yang, Z. (2014). The impact of the rate prior on Bayesian estimation of divergence times with multiple Loci. Syst. Biol. *63*, 555–565.

57. Zhu, T., Dos Reis, M., and Yang, Z. (2015). Characterization of the uncertainty of divergence time estimation under relaxed molecular clock models using multiple loci. Syst. Biol. *64*, 267–280.

58. Dunn, C.W., Giribet, G., Edgecombe, G.D., and Hejnol, A. (2014). Animal phylogeny and its evolutionary implications. Annu. Rev. Ecol. Evol. Syst. *45*, 371–395.

59. Darwin, C. (1859). On the Origin of Species (John Murray).

60. Davidson, E.H., and Erwin, D.H. (2006). Gene regulatory networks and the evolution of animal body plans. Science *311*, 796–800.

61. Erwin, D.H. (2011). Evolutionary uniformitarianism. Dev. Biol. *357*, 27–34.

62. Maloof, A.C., Rose, C.V., Beach, R., Samuels, B.M., Calmet, C.C., Erwin, D.H., Poirer, G.R., Yao, N., and Simons, F.J. (2010). Possible animal-body fossils in pre-Marinoan limestones from South Australia. Nat. Geosci. *3*, 653–659.

63. Budd, G.E., and Jensen, S. (2000). A critical reappraisal of the fossil record of the bilaterian phyla. Biol. Rev. Camb. Philos. Soc. *75*, 253–295.

64. Runnegar, B. (1982). The Cambrian Explosion - animals or fossils. J. Geol. Soc. Aust. *29*, 395–411.

65. Höhna, S., Stadler, T., Ronquist, F., and Britton, T. (2011). Inferring speciation and extinction rates under different sampling schemes. Mol. Biol. Evol. *28*, 2577–2589.

66. Lartillot, N., and Philippe, H. (2004). A Bayesian mixture model for across-site heterogeneities in the amino-acid replacement process. Mol. Biol. Evol. *21*, 1095–1109.

67. Rota-Stabelli, O., Daley, A.C., and Pisani, D. (2013). Molecular timetrees reveal a Cambrian colonization of land and a new scenario for ecdysozoan evolution. Curr. Biol. *23*, 392–398.

68. Brasier, M.D., Antcliffe, J.B., and Callow, R. (2011). Evolutionary trends in remarkable fossil preservation across the Ediacaran–Cambrian transition and the impact of Metazoan mixing. In Taphonomy: Bias and Process through Time, P.A. Allison, and D.J. Bottjer, eds. (Springer), pp. 519–567.

69. Pyron, R.A. (2011). Divergence time estimation using fossils as terminal taxa and the origins of Lissamphibia. Syst. Biol. *60*, 466–481.

70. Ronquist, F., Klopfstein, S., Vilhelmsen, L., Schulmeister, S., Murray, D.L., and Rasnitsyn, A.P. (2012). A total-evidence approach to dating with fossils, applied to the early radiation of the hymenoptera. Syst. Biol. *61*, 973–999.

71. dos Reis, M., Inoue, J., Hasegawa, M., Asher, R.J., Donoghue, P.C.J., and Yang, Z. (2012). Phylogenomic datasets provide both precision and accuracy in estimating the timescale of placental mammal phylogeny. Proc. Biol. Sci. *279*, 3491–3500.

72. Dos Reis, M., and Yang, Z. (2013). The unbearable uncertainty of Bayesian divergence time estimation. J. Syst. Evol. *51*, 30–43.

73. O'Reilly, J., dos Reis, M., and Donoghue, P.C.J. (2015). Dating tips for divergence time estimation. Trends Genet. Published online October 5, 2015. http://dx.doi.org/10.1016/j.tig.2015.08.001.

74. Heath, T.A., Huelsenbeck, J.P., and Stadler, T. (2014). The fossilized birth-death process for coherent calibration of divergence-time estimates. Proc. Natl. Acad. Sci. USA *111*, E2957–E2966.

75. Wilkinson, R.D., Steiper, M.E., Soligo, C., Martin, R.D., Yang, Z., and Tavaré, S. (2011). Dating primate divergences through an integrated analysis of palaeontological and molecular data. Syst. Biol. *60*, 16–31.

76. Marshall, C.R. (1997). Confidence intervals on stratigraphic ranges with nonrandom distributions of fossil horizons. Paleobiology *23*, 165–173.

77. Löytynoja, A., and Goldman, N. (2005). An algorithm for progressive multiple alignment of sequences with insertions. Proc. Natl. Acad. Sci. USA *102*, 10557–10562.

78. Castresana, J. (2000). Selection of conserved blocks from multiple alignments for their use in phylogenetic analysis. Mol. Biol. Evol. *17*, 540–552.

79. Benton, M.J., Donoghue, P.C.J., and Asher, R.J. (2009). Calibrating and constraining molecular clocks. In The Timetree of Life, B.S. Hedges, and S. Kumar, eds. (Oxford University Press), pp. 35–86.

80. Benton, M.J., Donoghue, P.C.J., Asher, R.J., Friedman, F., Near, T.J., and Vinther, J. (2015). Constraints on the timescale of animal evolutionary history. Palaeontologia Electronica, *18.1.1FC*, 1–107.

81. Benton, M.J., and Donoghue, P.C.J. (2007). Paleontological evidence to date the tree of life. Mol. Biol. Evol. *24*, 26–53.

82. Donoghue, P.C., and Benton, M.J. (2007). Rocks and clocks: calibrating the Tree of Life using fossils and molecules. Trends Ecol. Evol. *22*, 424–431.

83. dos Reis, M., and Yang, Z. (2011). Approximate likelihood calculation on a phylogeny for Bayesian estimation of divergence times. Mol. Biol. Evol. *28*, 2161–2172.

84. Le, S.Q., and Gascuel, O. (2008). An improved general amino acid replacement matrix. Mol. Biol. Evol. *25*, 1307–1320.

85. Yang, Z. (1994). Maximum likelihood phylogenetic estimation from DNA sequences with variable rates over sites: approximate methods. J. Mol. Evol. *39*, 306–314.

Current Biology

Supplemental Information

Uncertainty in the Timing of Origin of Animals and the Limits of Precision in Molecular Timescales

Mario dos Reis, Yuttapong Thawornwattana, Konstantinos Angelis, Maximilian J Telford, Philip C.J. Donoghue, and Ziheng Yang

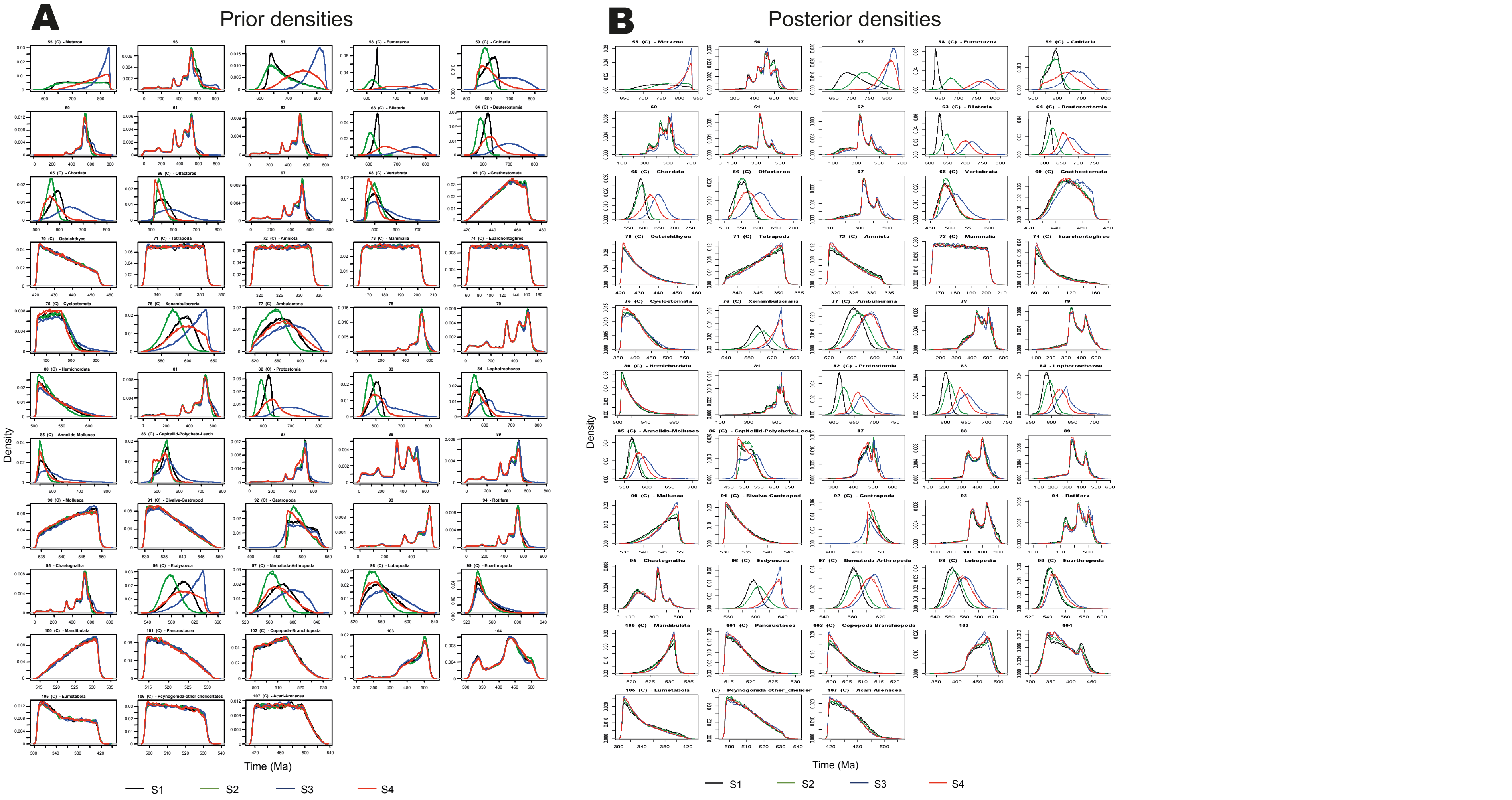

Figure S1.

Marginal prior (A) and marginal posterior (B) densities of divergence times for all nodes in the tree for the four different calibration strategies (Figure 1 in the main text). Nodes are numbered as in Figure 6 in the main text.

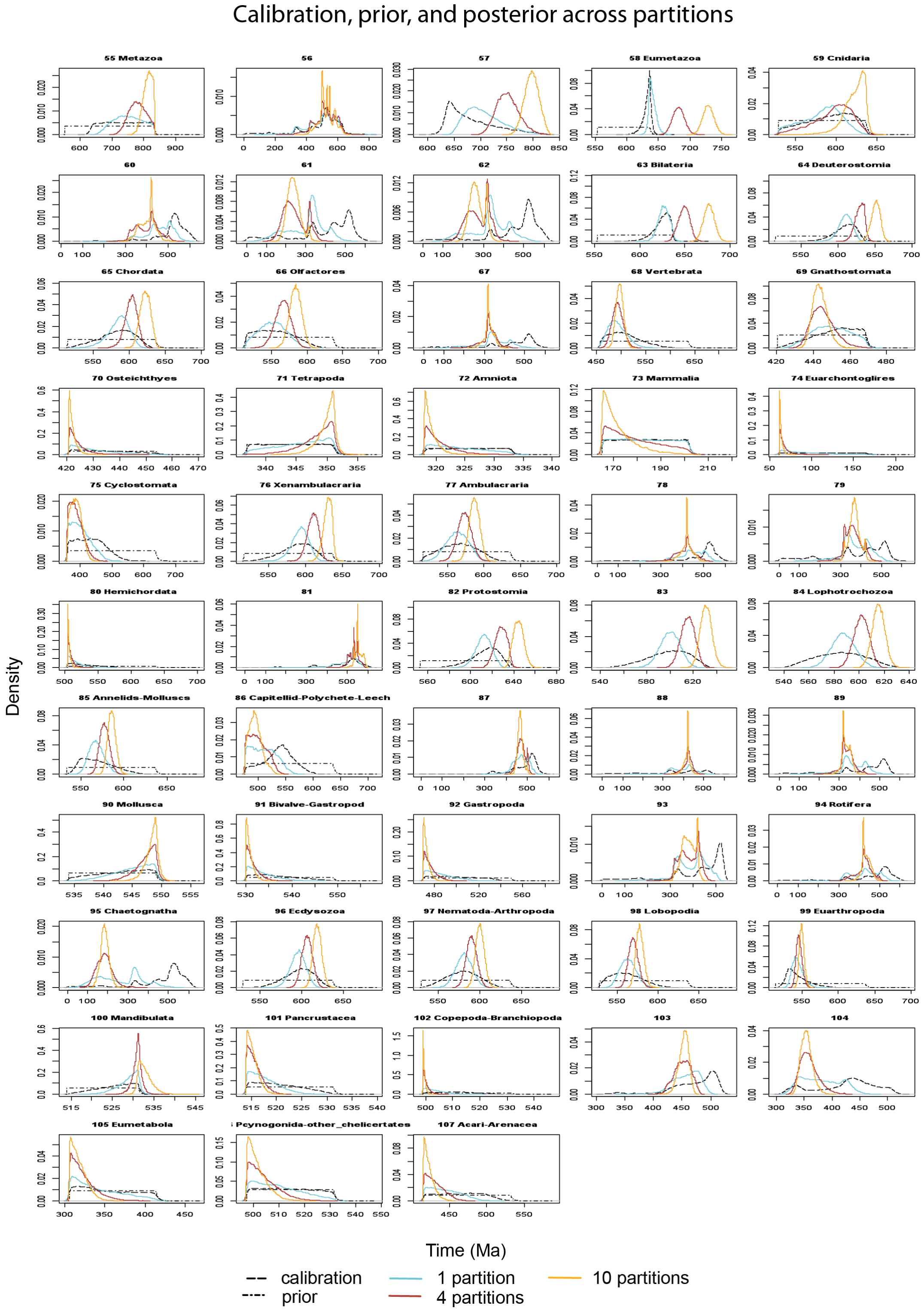

Figure S2.
Calibration, marginal prior and marginal posterior densities for various partitioning strategies. Nodes are numbered as in Figure 6 in the main text.

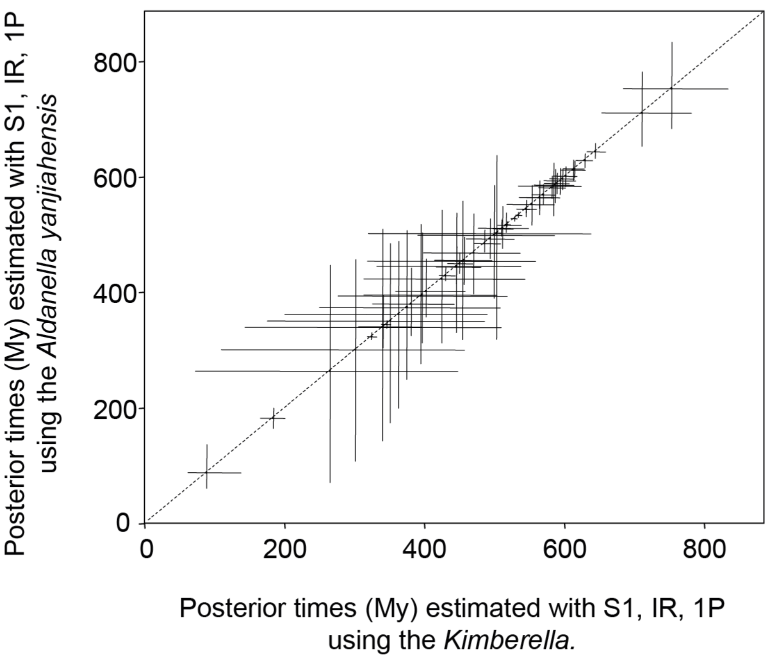

Figure S3.
Estimates of divergence times under calibration strategy 1, where the fossil *Kimberella* provides the minimum age constraint on the root, vs. estimates under strategy 1b, where *Aldanella* provides the minimum constraint instead. This extends Figure 2 in the main text.

Table S1. Fossil calibration densities constructed from the minimum and maximum contrains used in this study.

Node	Crown group	Min	Max	Strategy 1	Strategy 2	Strategy 3	Strategy 4
**55**	Metazoa	552.85	833	B(5.5285,8.33,0.001,0.001)	B(5.5285,8.33,0.001,0.001)	***B(6.349,8.33,0.001,0.001)***	***B(6.349,8.33,0.001,0.001)***
**58**	Eumetazoa	552.85	636.1	B(5.5285,6.361,0.001,0.025)	***SN(5.6,0.34,7)***	***L(5.5285,0,10,0.001)***	***L(5.5285,0,0.1,0.001)***
**59**	Cnidaria	529	636.1	B(5.29,6.361,0.001,0.025)	***SN(5.38,0.44,7)***	***L(5.29,0,10,0.001)***	***L(5.29,0,0.1,0.001)***
**63**	Bilateria	552.85	636.1	B(5.5285,6.361,0.001,0.025)	***SN(5.6,0.34,7)***	***L(5.5285,0,10,0.001)***	***L(5.5285,0,0.1,0.001)***
**64**	Deuterostomia	515.5	636.1	B(5.155,6.361,0.001,0.025)	***SN(5.255,0.5,7)***	***L(5.155,0,10,0.001)***	***L(5.155,0,0.1,0.001)***
**65**	Chordata	514	636.1	B(5.14,6.361,0.001,0.025)	***SN(5.25,0.5,7)***	***L(5.14,0,10,0.001)***	***L(5.14,0,0.1,0.001)***
**66**	Olfactores	514	636.1	B(5.14,6.361,0.001,0.025)	***SN(5.25,0.5,7)***	***L(5.14,0,10,0.001)***	***L(5.14,0,0.1,0.001)***
**68**	Vertebrata	457.5	636.1	B(4.575,6.361,0.001,0.025)	***SN(4.7,0.75,9)***	***L(4.575,0,10,0.001)***	***L(4.575,0,0.1,0.001)***
69	Gnathostomata	420.7	468.4	B(4.207,4.684,0.001,0.025)	B(4.207,4.684,0.001,0.025)	B(4.207,4.684,0.001,0.025)	B(4.207,4.684,0.001,0.025)
70	Osteichthyes	420.7	453.7	B(4.207,4.537,0.001,0.025)	B(4.207,4.537,0.001,0.025)	B(4.207,4.537,0.001,0.025)	B(4.207,4.537,0.001,0.025)
71	Tetrapoda	337	351	B(3.37,3.51,0.001,0.025)	B(3.37,3.51,0.001,0.025)	B(3.37,3.51,0.001,0.025)	B(3.37,3.51,0.001,0.025)
72	Amniota	318	332.9	B(3.18,3.329,0.001,0.025)	B(3.18,3.329,0.001,0.025)	B(3.18,3.329,0.001,0.025)	B(3.18,3.329,0.001,0.025)
73	Mammalia	164.9	201.5	B(1.649,2.015,0.001,0.025)	B(1.649,2.015,0.001,0.025)	B(1.649,2.015,0.001,0.025)	B(1.649,2.015,0.001,0.025)
74	Euarchontoglires	61.6	164.6	B(0.616,1.646,0.001,0.025)	B(0.616,1.646,0.001,0.025)	B(0.616,1.646,0.001,0.025)	B(0.616,1.646,0.001,0.025)
75	Cyclostomata	358.5	636.1	B(3.585,6.361,0.001,0.025)	B(3.585,6.361,0.001,0.025)	B(3.585,6.361,0.001,0.025)	B(3.585,6.361,0.001,0.025)
76	Xenambulacraria	515.5	636.1	B(5.155,6.361,0.001,0.025)	B(5.155,6.361,0.001,0.025)	B(5.155,6.361,0.001,0.025)	B(5.155,6.361,0.001,0.025)
77	Ambulacraria	515.5	636.1	B(5.155,6.361,0.001,0.025)	B(5.155,6.361,0.001,0.025)	B(5.155,6.361,0.001,0.025)	B(5.155,6.361,0.001,0.025)
80	Hemichordata	504.5	636.1	B(5.045,6.361,0.001,0.025)	B(5.045,6.361,0.001,0.025)	B(5.045,6.361,0.001,0.025)	B(5.045,6.361,0.001,0.025)
**82**	Protostomia	552.85	636.1	B(5.5285,6.361,0.001,0.025)	***SN(5.6,0.34,7)***	***L(5.5285,0,10,0.001)***	***L(5.5285,0,0.1,0.001)***
**85**	Annelids-Molluscs	534	636.1	B(5.34,6.361,0.001,0.025)	***SN(5.41,0.43,9)***	***L(5.34,0,10,0.001)***	***L(5.34,0,0.1,0.001)***
**86**	Capitellid-Polychete-leech	476.5	636.1	B(4.765,6.361,0.001,0.025)	***SN(4.86,0.68,10)***	***L(4.765,0,10,0.001)***	***L(4.765,0,0.1,0.001)***
90	Mollusca	534	549	B(5.34,5.49,0.001,0.025)	B(5.34,5.49,0.001,0.025)	B(5.34,5.49,0.001,0.025)	B(5.34,5.49,0.001,0.025)
91	Bivalve-Gastropod	530	549	B(5.30,5.49,0.001,0.025)	B(5.30,5.49,0.001,0.025)	B(5.30,5.49,0.001,0.025)	B(5.30,5.49,0.001,0.025)
**92**	Gastropoda	470.2	549	B(4.702,5.49,0.001,0.025)	***SN(4.75,0.33,9)***	***L(4.702,0,10,0.001)***	***L(4.702,0,0.1,0.001)***
96	Ecdysozoa	528.82	636.1	B(5.2882,6.361,0.001,0.025)	B(5.2882,6.361,0.001,0.025)	B(5.2882,6.361,0.001,0.025)	B(5.2882,6.361,0.001,0.025)

**97**	Nematoda-Arthropoda	528.82	636.1	B(5.2882,6.361,0.001,0.025)	***SN(5.38,0.44,7)***	***L(5.2882,0,10,0.001)***	***L(5.2882,0,0.1,0.001)***
98	Lobopodia	528.82	636.1	B(5.2882,6.361,0.001,0.025)	B(5.2882,6.361,0.001,0.025)	B(5.2882,6.361,0.001,0.025)	B(5.2882,6.361,0.001,0.025)
**99**	Euarthropoda	514	636.1	B(5.14,6.361,0.001,0.025)	***SN(5.22,0.52,9)***	***L(5.14,0,10,0.001)***	***L(5.14,0,0.1,0.001)***
100	Mandibulata	514	531.22	B(5.14,5.3122,0.001,0.025)	B(5.14,5.3122,0.001,0.025)	B(5.14,5.3122,0.001,0.025)	B(5.14,5.3122,0.001,0.025)
101	Pancrustacea	514	531.22	B(5.14,5.3122,0.001,0.025)	B(5.14,5.3122,0.001,0.025)	B(5.14,5.3122,0.001,0.025)	B(5.14,5.3122,0.001,0.025)
102	Copepoda-Branchiopoda	499	531.22	B(4.99,5.3122,0.001,0.025)	B(4.99,5.3122,0.001,0.025)	B(4.99,5.3122,0.001,0.025)	B(4.99,5.3122,0.001,0.025)
105	Eumetabola	305.5	413.6	B(3.055,4.136,0.001,0.025)	B(3.055,4.136,0.001,0.025)	B(3.055,4.136,0.001,0.025)	B(3.055,4.136,0.001,0.025)
106	Pycnogonida-other chelicertates	497.5	531.22	B(4.975,5.3122,0.001,0.025)	B(4.975,5.3122,0.001,0.025)	B(4.975,5.3122,0.001,0.025)	B(4.975,5.3122,0.001,0.025)
107	Acari-Arenacea	416	531.22	B(4.16,5.3122,0.001,0.025)	B(4.16,5.3122,0.001,0.025)	B(4.16,5.3122,0.001,0.025)	B(4.16,5.3122,0.001,0.025)

Note: B(t_L, t_U, p_L, p_U) means the node age has a soft uniform distribution between a minimum time t_L and a maximum time t_U, with probabilities p_L and p_U that the age is outside the bounds. SN(t, a, b) means the node age has a skew-normal distribution with location t, scale a, and shape b. L(t_L, p, c, p_L) means that the node age has a Cauchy distribution truncated on the left at t_L, with mode parameter p, tail parameter c, and probability p_L that the node age is younger than the minimum bound. Nodes and calibration densities that are different among the calibration strategies are indicated with bold typeface and italics. Fossil minima and maxima are from a draft version of Benton et al. (2015, *Paleontologica Electronica*, 18.1.1FC; 1–106). Note there are slight discrepancies between some of the minima and maxima above and the values in the final version of Benton et al. The discrepancies are around 2 My or less and are unimportant given the large width (max – min) of the calibrations. Node numbers as in Figure 6 in the main text.

Table S2. Minimum and maximum fossil constraints and 95% HPD CI of prior divergence times (Ma) for all metazoan clades under the four calibration strategies.

Node	Crown group	Min	Max	S1, IR, 1P		S2, IR, 1P		S3, IR, 1P		S4, IR, 1P	
55	Metazoa	552.85	833	641.3	832.6	629.5	833.2	757.5	833.5	689.0	833.3
56				123.1	679.0	115.6	664.5	160.0	826.9	129.8	768.3
57				622.4	778.9	599.9	777.8	738.2	832.5	662.6	826.7
58	Eumetazoa	552.85	636.1	616.6	642.7	589.5	658.7	717.4	830.5	629.4	806.7
59	Cnidaria	529	636.1	538.8	634.8	536.7	615.8	543.5	795.1	529.0	687.5
60				371.8	630.3	373.7	609.4	332.1	702.7	336.6	625.1
61				49.2	577.5	58.7	567.3	53.8	616.1	50.1	572.1
62				139.9	626.1	135.2	602.5	108.4	704.4	121.3	631.0
63	Bilateria	552.85	636.1	605.4	637.6	579.6	635.6	677.0	817.9	598.3	751.5
64	Deuterostomia	515.5	636.1	581.0	633.7	558.2	618.9	618.4	785.4	564.5	695.3
65	Chordata	514	636.1	535.2	623.1	532.1	596.3	546.0	749.5	519.0	630.1
66	Olfactores	514	636.1	513.9	598.1	518.1	576.0	506.8	696.9	513.9	584.2
67				107.8	592.6	112.6	573.8	110.6	641.8	89.7	570.1
68	Vertebrata	457.5	636.1	457.4	565.4	464.6	545.7	451.9	633.4	457.4	532.4
69	Gnathostomata	420.7	468.4	429.6	469.6	430.1	470.1	429.6	469.6	429.8	469.5
70	Osteichthyes	420.7	453.7	420.7	451.2	420.7	451.4	420.7	451.2	420.6	451.2
71	Tetrapoda	337	351	337.2	350.9	337.2	350.9	337.2	350.9	337.4	351.0
72	Amniota	318	332.9	318.3	332.9	318.2	332.7	318.3	332.8	318.1	332.6
73	Mammalia	164.9	201.5	165.6	201.2	165.5	201.2	165.9	201.5	165.6	201.2
74	Euarchontoglires	61.6	164.6	63.2	163.6	63.9	163.8	63.5	163.6	63.3	163.5
75	Cyclostomata	358.5	636.1	358.1	509.9	358.3	500.6	358.1	539.8	358.2	491.4
76	Xenambulacraria	515.5	636.1	547.0	625.3	534.4	605.8	561.6	643.2	548.2	638.8
77	Ambulacraria	515.5	636.1	519.3	605.8	516.1	586.7	526.8	630.5	517.6	616.2
78				335.9	592.6	338.9	578.2	331.8	607.7	334.7	595.2
79				45.3	556.6	51.0	552.9	34.7	550.4	50.1	556.5
80	Hemichordata	504.5	636.1	504.2	577.4	504.2	563.8	504.3	593.7	504.3	584.4
81				133.2	613.3	136.6	595.9	140.3	634.7	146.0	628.0
82	Protostomia	552.85	636.1	587.2	634.4	567.3	619.5	620.8	786.0	573.6	693.7
83				563.3	628.8	552.0	608.3	572.3	756.5	551.3	655.7
84				548.9	619.4	545.1	597.7	549.2	726.2	543.4	631.5
85	Annelids-Molluscs	534	636.1	539.4	605.5	539.8	582.6	539.6	693.8	536.4	601.8
86	Capitellid-Polychete-Leech	476.5	636.1	476.7	581.5	487.2	566.3	471.7	642.7	476.5	565.5
87				321.4	570.8	323.4	557.4	321.0	607.6	315.4	559.4
88				39.9	542.9	41.3	538.2	37.3	545.9	37.1	534.8
89				85.5	567.6	90.9	557.9	70.6	591.6	82.0	558.7
90	Mollusca	534	549	535.2	549.3	535.2	549.3	535.4	549.5	535.0	549.2
91	Bivalve-Gastropod	530	549	530.0	545.2	530.0	544.7	530.0	545.3	530.0	544.9
92	Gastropoda	470.2	549	470.2	532.7	472.3	527.0	460.7	536.8	470.1	528.2
93				106.6	544.9	106.7	545.5	100.6	544.4	108.4	545.5
94				134.0	613.7	134.2	592.0	104.0	646.5	126.1	612.9
95				147.7	623.1	140.4	600.8	110.5	685.0	137.9	634.0
96	Ecdysozoa	528.82	636.1	562.8	627.6	551.4	607.6	575.8	641.6	560.3	638.2
97	Nematoda-Arthropoda	528.82	636.1	543.0	614.7	539.7	591.8	551.7	634.0	538.2	617.8
98	Lobopodia	528.82	636.1	529.7	595.0	529.3	577.1	531.7	613.4	528.8	594.2
99	Euarthropoda	514	636.1	520.8	574.9	522.3	560.6	521.3	588.1	519.5	567.8
100	Mandibulata	514	531.22	517.6	532.0	517.6	531.8	517.6	531.9	517.3	531.7
101	Pancrustacea	514	531.22	514.0	528.2	514.0	528.1	514.0	528.3	514.0	528.0
102	Copepoda-	499	531.22	499.0	522.3	499.0	522.1	499.0	522.4	499.0	522.0

	Branchiopoda										
103				388.4	524.7	393.1	525.3	386.4	524.9	396.9	524.7
104				321.7	507.1	323.4	509.4	319.4	505.1	322.4	506.8
105	Eumetabola	305.5	413.6	305.4	409.2	305.4	409.7	305.5	409.6	305.4	409.1
106	Pcynogonida-other chelicertates	497.5	531.22	497.5	529.9	497.5	529.8	497.6	530.1	497.5	529.7
107	Acari-Arenacea	416	531.22	415.9	509.7	416.0	509.8	416.0	509.3	416.0	509.6

Note: Prior times are 95% HPD intervals estimated by running MCMCTree without sequence data under the four calibration strategies. S1–S4. IR: Independent-rates model. 1P: The 203 proteins analysed as a single partition. Node numbers are as in Figure 6 in the main text.

Table S3. Minimum and maximum fossil constraints and 95% HPD CI of posterior divergence times (Ma) for all metazoan clades under the four calibration strategies.

Node	Crown group	Min	Max	S1, IR, 1P		S2, IR, 1P		S3, IR, 1P		S4, IR, 1P	
55	Metazoa	552.85	833	680.6	832.7	716.2	833.4	795.2	833.6	780.0	833.5
56				314.6	639.9	318.6	646.6	319.2	670.6	319.2	661.1
57				649.2	776.7	686.1	805.5	779.5	832.2	761.6	831.4
58	Eumetazoa	552.85	636.1	630.7	652.9	649.5	714.2	738.5	808.8	715.4	798.7
59	Cnidaria	529	636.1	533.3	620.5	537.7	631.9	583.8	760.0	531.5	715.7
60				318.9	554.4	319.3	550.0	350.7	637.9	319.6	591.2
61				110.4	458.5	118.1	452.7	126.1	475.4	129.2	461.8
62				125.5	488.1	133.8	485.5	188.3	542.2	167.2	519.5
63	Bilateria	552.85	636.1	615.1	637.8	624.2	672.3	685.4	759.2	666.4	736.4
64	Deuterostomia	515.5	636.1	593.7	627.9	598.0	649.6	643.7	721.7	625.9	695.3
65	Chordata	514	636.1	555.4	611.3	558.1	622.2	600.5	693.3	568.6	662.6
66	Olfactores	514	636.1	516.6	583.6	524.3	588.0	548.2	656.1	521.8	618.6
67				167.9	480.9	193.2	485.3	236.2	526.3	203.9	486.4
68	Vertebrata	457.5	636.1	459.6	527.9	467.1	527.6	469.2	564.7	461.8	533.5
69	Gnathostomata	420.7	468.4	432.9	468.7	433.9	468.6	435.9	469.4	433.8	468.4
70	Osteichthyes	420.7	453.7	420.6	444.1	420.6	443.9	420.6	443.6	420.6	441.9
71	Tetrapoda	337	351	338.3	351.4	338.4	351.5	338.8	351.6	338.7	351.6
72	Amniota	318	332.9	318.0	331.4	318.0	331.1	318.0	330.7	318.0	330.7
73	Mammalia	164.9	201.5	165.1	200.7	164.9	200.5	164.9	200.6	165.0	200.5
74	Euarchontoglires	61.6	164.6	61.4	140.2	61.4	135.3	61.4	127.6	61.3	128.4
75	Cyclostomata	358.5	636.1	358.1	458.0	358.1	455.8	358.1	469.1	358.1	453.0
76	Xenambulacraria	515.5	636.1	569.8	614.5	575.9	632.2	606.4	646.4	600.6	644.4
77	Ambulacraria	515.5	636.1	534.6	591.3	538.5	603.5	554.8	620.1	552.7	618.8
78				330.6	537.8	334.3	541.3	348.9	550.0	343.3	548.1
79				250.6	507.0	266.4	509.1	285.6	510.8	277.5	508.1
80	Hemichordata	504.5	636.1	504.2	537.6	504.2	540.0	504.1	545.6	504.2	546.2
81				378.5	585.8	404.9	594.0	421.7	605.3	420.1	605.0
82	Protostomia	552.85	636.1	598.0	626.4	603.6	647.5	644.4	712.3	632.2	690.5
83				582.7	616.2	587.6	633.1	620.3	693.2	610.6	672.4
84				570.0	605.7	573.7	618.3	596.5	671.0	588.8	649.1
85	Annelids-Molluscs	534	636.1	552.3	586.1	554.1	591.7	564.2	630.1	559.5	611.9
86	Capitellid-Polychete-Leech	476.5	636.1	476.3	548.1	480.9	550.9	468.3	573.6	476.4	550.0
87				398.5	536.0	407.4	534.2	413.1	548.3	406.2	533.6
88				310.5	501.1	312.3	489.6	315.3	499.9	312.1	481.2
89				201.6	487.0	220.6	485.2	231.3	482.9	226.7	473.5
90	Mollusca	534	549	538.4	549.6	539.1	549.7	540.8	550.0	540.5	550.0
91	Bivalve-	530	549	530.0	539.1	530.0	538.6	530.0	538.2	530.0	538.3

92	Gastropod Gastropoda	470.2	549	470.0	508.3	470.3	506.2	450.8	505.3	470.1	500.9
93				265.0	516.5	285.1	512.1	300.3	505.4	291.9	507.6
94				310.6	541.4	314.2	538.2	319.4	549.4	318.5	544.6
95				72.7	452.4	84.3	447.5	93.3	454.7	88.8	447.7
96	Ecdysozoa	528.82	636.1	577.8	613.2	581.9	627.1	610.1	644.5	602.6	641.6
97	Nematoda-Arthropoda	528.82	636.1	561.4	599.8	563.8	608.3	583.9	628.6	577.8	625.0
98	Lobopodia	528.82	636.1	545.1	582.8	547.8	588.5	558.5	606.1	554.7	602.0
99	Euarthropoda	514	636.1	530.8	559.4	531.9	560.7	535.4	571.0	534.5	567.1
100	Mandibulata	514	531.22	523.4	532.3	524.0	532.3	525.2	532.6	525.0	532.4
101	Pancrustacea	514	531.22	514.0	522.8	514.0	522.3	514.0	521.8	514.0	521.9
102	Copepoda-Branchiopoda	499	531.22	499.0	510.1	498.9	509.2	498.9	508.0	498.9	508.3
103				414.4	496.1	414.2	493.6	418.0	490.3	417.6	491.5
104				324.8	441.5	325.3	438.8	327.0	433.4	327.6	435.5
105	Eumetabola	305.5	413.6	305.3	396.8	305.3	393.1	305.2	387.2	305.2	388.3
106	Pcynogonida-other chelicertates	497.5	531.22	497.5	526.1	497.5	525.8	497.5	526.9	497.5	526.4
107	Acari-Arenacea	416	531.22	415.9	479.9	415.8	477.5	415.9	474.0	415.8	474.4

Note: Posterior times are the 95% HPD interval, estimated with MCMCTree under the $LG+G_4+F$ model, using four calibration strategies S1–S4. IR: Independent-rates model. 1P: The 203 proteins analysed as a single partition. Node numbers are as in Figure 6 in the main text.

Table S4. 95% HPD CI of posterior divergence times (Ma) for all metazoan clades under various partitioning strategies.

Node	Crown group	S1, IR, 1P		S2, IR, 2P		S3, IR, 4P		S4, IR, 5P		S4, IR, 10P	
55	Metazoa	680.6	832.7	701.0	831.2	736.9	832.6	748.9	832.3	786.8	833.5
56		314.6	639.9	326.2	632.6	387.9	639.0	413.3	639.2	440.3	631.0
57		649.2	776.7	674.7	781.6	712.5	794.3	726.8	798.5	771.7	823.1
58	Eumetazoa	630.7	652.9	638.9	669.0	664.2	699.9	677.5	711.0	712.2	746.2
59	Cnidaria	533.3	620.5	532.7	620.2	548.7	635.6	559.0	637.4	596.2	641.7
60		318.9	554.4	315.9	531.3	315.7	501.8	310.6	487.4	335.4	469.1
61		110.4	458.5	119.5	388.5	154.1	349.4	159.3	336.3	186.2	320.4
62		125.5	488.1	155.3	432.4	176.7	365.6	187.8	358.0	207.0	331.1
63	Bilateria	615.1	637.8	623.1	643.3	636.6	660.0	646.4	666.5	665.6	688.3
64	Deuterostomia	593.7	627.9	602.2	630.1	617.0	640.5	624.2	644.0	639.5	662.3
65	Chordata	555.4	611.3	567.9	611.3	586.2	619.3	593.4	621.6	609.0	635.7
66	Olfactores	516.6	583.6	527.9	584.5	544.0	586.5	552.0	589.6	568.0	600.0
67		167.9	480.9	215.4	444.5	233.7	391.3	246.4	383.9	274.6	371.0
68	Vertebrata	459.6	527.9	464.5	520.8	472.3	515.6	475.4	514.4	483.3	512.9
69	Gnathostomata	432.9	468.7	432.5	464.7	433.6	457.4	434.8	456.2	436.2	451.3
70	Osteichthyes	420.6	444.1	420.6	437.8	420.6	430.6	420.6	428.8	420.6	425.0
71	Tetrapoda	338.3	351.4	339.8	351.7	342.7	351.9	343.8	352.0	346.5	352.1
72	Amniota	318.0	331.4	318.0	329.3	318.0	325.2	318.0	323.9	318.0	321.5
73	Mammalia	165.1	200.7	164.9	200.0	164.8	197.8	164.8	196.4	164.8	186.5
74	Euarchontoglires	61.4	140.2	61.3	102.8	61.3	76.8	61.3	73.2	61.3	67.3
75	Cyclostomata	358.1	458.0	358.3	442.2	358.1	426.1	358.2	420.4	358.3	416.5
76	Xenambulacraria	569.8	614.5	580.7	615.2	595.1	623.7	601.7	626.6	617.6	639.9

77	Ambulacraria	534.6	591.3	542.9	588.2	555.1	591.4	559.6	592.3	572.6	600.1
78		330.6	537.8	331.9	516.0	341.2	488.9	348.9	481.5	367.8	469.9
79		250.6	507.0	268.9	468.2	296.2	445.1	304.2	436.2	317.9	422.4
80	Hemichordata	504.2	537.6	504.1	525.8	504.1	517.5	504.1	515.5	504.1	511.4
81		378.5	585.8	441.9	575.7	492.5	578.8	497.3	576.4	526.9	588.8
82	Protostomia	598.0	626.4	605.5	628.4	617.6	637.8	624.1	640.3	635.3	653.5
83		582.7	616.2	591.8	618.1	603.6	626.5	609.7	628.6	621.2	640.5
84		570.0	605.7	578.4	605.9	590.2	613.5	595.6	615.6	605.7	625.4
85	Annelids-Molluscs	552.3	586.1	559.2	585.3	567.2	588.8	570.6	590.3	577.4	595.1
86	Capitellid-Polychete-Leech	476.3	548.1	476.3	536.4	476.3	528.3	476.3	526.4	476.3	517.5
87		398.5	536.0	421.0	517.4	435.6	507.6	439.3	505.6	439.2	493.5
88		310.5	501.1	320.7	469.9	362.9	465.2	371.9	462.2	384.0	446.6
89		201.6	487.0	248.1	452.6	265.9	417.3	272.5	401.6	295.2	379.2
90	Mollusca	538.4	549.6	540.8	549.8	543.4	549.9	544.3	550.0	545.8	550.3
91	Bivalve-Gastropod	530.0	539.1	530.0	536.5	530.0	534.2	530.0	533.7	530.0	532.6
92	Gastropoda	470.0	508.3	470.1	497.9	470.0	487.4	470.0	484.6	470.0	478.8
93		265.0	516.5	304.5	486.8	313.2	452.3	314.3	444.6	324.8	431.8
94		310.6	541.4	317.1	512.1	344.0	502.9	349.3	489.6	394.2	481.2
95		72.7	452.4	90.5	350.9	110.4	254.0	122.5	248.1	140.6	225.2
96	Ecdysozoa	577.8	613.2	585.3	613.2	594.3	618.4	599.6	620.5	608.8	628.9
97	Nematoda-Arthropoda	561.4	599.8	568.6	598.4	577.7	602.4	581.6	604.0	589.8	610.4
98	Lobopodia	545.1	582.8	551.8	580.3	558.4	581.2	561.8	582.9	568.5	587.0
99	Euarthropoda	530.8	559.4	534.8	555.5	538.9	554.5	540.1	554.3	543.3	556.2
100	Mandibulata	523.4	532.3	526.2	532.6	528.4	533.1	528.9	533.4	530.3	536.1
101	Pancrustacea	514.0	522.8	514.0	520.4	514.0	518.5	514.0	518.0	514.0	517.5
102	Copepoda-Branchiopoda	499.0	510.1	498.9	505.8	498.9	502.5	498.9	501.8	498.9	500.5
103		414.4	496.1	420.1	485.7	423.7	476.8	426.1	474.9	435.2	468.1
104		324.8	441.5	328.0	424.0	330.4	392.4	332.8	387.2	334.8	374.4
105	Eumetabola	305.3	396.8	305.3	378.9	305.2	352.7	305.3	347.2	305.3	335.8
106	Pcynogonida-other chelicertates	497.5	526.1	497.4	520.5	497.4	514.7	497.4	512.5	497.4	509.1
107	Acari-Arenacea	415.9	479.9	415.9	466.3	415.8	453.2	415.7	448.4	415.8	436.4

Note: Posterior times are the 95% HPD interval, estimated with MCMCTree v4.8 under the LG+G_4+F model, using calibration strategies 1 and different partition strategies. 1P: The 203 proteins analysed as a single partition. 2P, 4P, 5P , 10P: The proteins are grouped into 2, 4, 5, 10 partitions according to their evolutionary rates. Node numbers are as in Figure 6 in the main text.

Supplemental Experimental Procedures

Data assembly

Two independent molecular data sets from [1] and [2] were updated and combined into a single amino acid alignment. Missing or incomplete proteins in the original alignments were updated with the non-redundant protein database from GenBank, with priority given to proteins from RefSeq. The protein sequence of each gene in each original alignment was used as a query for the BLASTp program. The best hit was accepted if the e-value was below 10^{-30} and the mismatches (excluding gaps) were lower than 5%. In addition, 5 new species (*Homo sapiens*, *Mus musculus*, *Ornithorhynchus anatinus*, *Tribolium castaneum* and *Caenorhabditis elegans*) were added to the data set in order to accommodate more calibration points. This was done in a similar manner as described above, but with the sequence of a closely related species according to [1] as a query instead.

For each gene, amino acid sequences of all species were aligned with PRANK [3]. The alignment gaps were removed using GBLOCKS [4] with the same setting as in [1]. All sub-alignments of individual genes were combined into a single alignment. This alignment contains 71 species with 38,577 amino acid positions from 203 nuclear coding genes (missing data 21.49%). This process recovered the original alignments but with extra species and sequences of genes previously missing or incomplete. Note that one gene was present in both data sets [1, 2], and hence was removed before the two data sets were combined. Further modifications to this alignment are described below.

Tree topology

The tree topology used is mainly according to [1] with some adjustments based on current knowledge. As the relationships among many taxa remain largely unresolved, 17 species were removed from the dataset in order to reduce the uncertainty in the topology. This resulted in a smaller alignment of the remaining 54 species (missing data 13.97%). The tree topology for these 54 species has 4 uncertain nodes that can be rearranged in three ways and one uncertain node that can be rearranged in two ways, giving $3^4 \times 2 = 162$ possible fully resolved trees. One of these trees was chosen (based on generally accepted positions) for the main analysis while the other 161 trees were used to assess the robustness of the time estimates to the various topologies.

Data partitioning

Two partitioning schemes were considered. First, the relative evolutionary rates among genes (evolutionary distances) were used for partitioning. Amino acid distance estimates for each gene were obtained from pairwise comparisons between *Strongylocentrotus purpuratus* and *Hydra magnipapillata* under the WAG+Γ_4+F model in CODEML v4.5 [5]. These two species were chosen because of their deep divergence time and because they have the most complete sequence data. For one missing gene of *Strongylocentrotus purpuratus*, the same gene of *Saccoglossus kowalevskii*, its close relative, was used instead. Because the divergence time is the same for all genes, the estimated distances can be used as a measure of the evolutionary rate. These distances were used to assign the 203 genes into different rate groups. Here, the numbers of rate categories (hence the number of partitions) considered were two, four, five, ten, as well as a single partition.

There is a possibility, however, that the rate estimated from these two chosen species may not be representative of the rates across branches, or the rates could be too varied to be used for partitioning. To address this issue, the suitability of this partitioning scheme was assessed by calculating the branch lengths of each partition using the WAG+Γ_4+F model. If the use of the rates is suitable in partitioning the data, the sum of the branch lengths (i.e. tree length) is expected to be approximately ordered from a partition with the lowest rate

category to one with the highest rate category. This was found to be the case.

Second, the data was divided into two partitions according to hydrophobicity, using the hydropathy index [6]. An average of the hydropathy index for each site in the alignment was calculated (gaps excluded). Then the site was classified as hydrophilic if the averaged hydropathy index was negative, otherwise it was classified as hydrophobic. Then the times were estimated with these two partitions under the LG+Γ_4+F model and again under the GTR+Γ_4+F model. For both models, the partitioning was virtually the same as the two-partition analysis according to rate and thus are not reported here.

Fossil Constraints

Thirty-four calibrations were derived mainly from ref [7] with updates from refs [8, 9]. The minimum ages were determined from the oldest certain record belonging to one of the two sister clades. These inferred minima are conservative and the actual origination time of a clade is likely to be older. The maximum ages were derived from the base of the youngest stratigraphic range or geological formation known not to contain any members of the clade of interest [10, 11]. Note that inferring a maximum date involves higher uncertainty and in most cases, the true origination dates are expected to be closer to the minimum constraint. A critical fossil is the Ediacaran *Kimberella* (552.85 Ma) which we interpret as a protostome, thus providing the minimum age constraint for Metazoa, Eumetazoa, Bilateria and Protostomia.

Fossil calibrations must be represented as statistical distributions mapped onto nodes. The choice of statistical distribution is subjective and may have a strong impact on estimated times [8, 12]. To assess the robustness of Metazoan divergences to calibration choice, we used four calibration strategies (Table S1):

(1) Strategy 1 (S1): The 34 calibrations are represented as uniform distributions between the min. and max. bounds. Bounds are soft, and we assign 0.1% and 2.5% tail probabilities that min. and max. bounds are violated (but we use 0.1% for both min. and max. bounds on the age of the root). A variation of S1 was also tested were the Cambrian snail *Aldanella* (532 Ma) was used instead of Kimberella to constrain the basal nodes. This change did not affect the results significantly.

(2) Strategy 2 (S2): 13 calibrations are represented as skewed-normal distributions. This was done for nodes for which the oldest ingroup fossil is thought to be very close to the actual parent node being calibrated. The parameters of the skew-normal (location, scale, shape) were thus chosen to provide a distribution with the mode near the minimum bound and the tail extending towards old ages, with the 0.3% and 97.5% quantiles of the distribution lying roughly at the equivalent minimum and maximum bounds from strategy 1. The remaining 21 nodes are as in strategy 1.

(3) Strategy 3 (S3): The same 13 nodes are calibrated using a truncated Cauchy distribution [12] with 0.1% left tail probability, with the mode of the distribution on the minimum bound, and with tail parameter equal to 10, leading to a long right tail for the distribution. No maximum bound is imposed on these nodes. The root node has an older minimum bound (634.9 Ma) accounting for alternative fossil interpretations.

(4) Strategy 4 (S4): Like strategy 3, but the tail parameter is 0.1 rather than 10, producing a truncated-Cauchy calibration with a much shorter tail. Note that the Cauchy is a heavy-tail distribution, and the tail thus extend further back in time than in the case of the skew-normal (S2).

Divergence time estimation

All molecular dating analyses were performed using the program MCMCtree in the PAML v4.8 package [5]. Analysis details are as follows:

(1) Priors for times

The time unit was set to 100 My. The prior on times was constructed using the fossil calibrations and the birth-death process [13]. The parameters of the birth-death process were set to $\lambda = \mu = 1$, $\rho = 0$. This gives a uniform kernel and hence represents a diffuse prior.

In theory, one should specify a joint prior distribution of all node ages (and of all rates) in a Bayesian dating analysis, which should summarize all information in the fossil record. However, specifying high-dimensional prior densities with strong correlation structures is a task too daunting. Instead, current dating programs allow the user to specify calibration densities on individual node ages, and then a truncation is applied to ensure that ancestral nodes are older than descendant nodes. In MCMCTREE, this truncated joint density is combined with the probability density for the non-calibrated nodes specified by the birth-death process to produce the effective prior, i.e. the prior used by the program. As a result, a marginal prior distribution used in MCMCTREE can be very different from the specified calibration density. To assess the truncation on marginal distributions in the marginal prior were obtained by running the MCMC without sequence data and compared with the calibration densities. In addition, comparing these marginal priors with the marginal posteriors for times allows the relative impact of the prior and the sequence information to be assessed. The marginal priors for all the nodes are shown in Figure S1A.

(2) Approximate likelihood calculation

Since the data set used in this study is large, the likelihood function for each data partition was calculated using the approximate likelihood method [14, 15]. First, the branch lengths were estimated in CODEML using the LG+Γ_4+F model [16, 17]. Next, the estimated branch lengths, the gradient and the Hessian matrix were used to compute the likelihood by the second-order Taylor approximation to the likelihood function [15]. This method greatly reduces the computational time in likelihood calculation compared to the exact method, which could take much longer for a data set of this size.

(3) Rate drift model and priors on rates

We used the independent rate model, which assumes that the rates for all branches are i.i.d. log-normal random variables [18]. The log-normal density is specified by two parameters, the mean of the rate μ (representing the overall rate) and the variance σ^2 of the log-rate (determining the degree of rate variation across branches). Here, the gamma-Dirichlet prior was used for both parameters [19]. The overall rate, μ, was assigned a gamma prior, G(2, 40), which is a diffuse prior with a mean of 0.05 (meaning 5×10^{-10} amino acid substitutions per site per year). This was derived from the average pairwise amino acid distances between the 203 proteins of *Hydra magnipapillata* and *Strongylocentrotus purpuratus* (0.29 substitutions/site) assuming a divergence time of 636.1 Ma, so that the mean rate is 0.29/6.361 = 0.46 ≈ 0.05. By fixing the shape parameter to 2, this gives the scale parameter as 40. The prior for σ^2 was set to G(1, 10). We also used the autocorrelated-rates model [18] to assess the effect of rate-drift model on time estimates. The same gamma priors on μ was used and the prior on σ^2 was set to G(1, 10).

The number of iterations, the burn-in and the sampling frequency were adjusted in test runs of the program. The step sizes of the proposals used in MCMC were adjusted such that the acceptance proportions were close to 0.3. In addition, at least two chains were run to ensure convergence. Convergence was assessed by comparing the posterior means and plotting the time series traces of the MCMC samples from the two independent runs. The

resulting posterior distribution from one of the two runs was summarized and the means and 95% HPD intervals were collected.

Data availability
The molecular sequence alignment and trees with fossil calibrations have been deposited in Figshare: DOI: 10.6084/m9.figshare.1525089.

References

S1. Philippe, H., Brinkmann, H., Copley, R.R., Moroz, L.L., Nakano, H., Poustka, A.J., Wallberg, A., Peterson, K.J., and Telford, M.J. (2011). Acoelomorph flatworms are deuterostomes related to Xenoturbella. Nature *470*, 255-225.

S2. Erwin, D.H., Laflamme, M., Tweedt, S.M., Sperling, E.A., Pisani, D., and Peterson, K.J. (2011). The Cambrian Conundrum: Early Divergence and Later Ecological Success in the Early History of Animals. Science *334*, 1091-1097.

S3. Löytynoja, A., and Goldman, N. (2005). An algorithm for progressive multiple alignment of sequences with insertions. Proc Natl Acad Sci USA *102*, 10557-10562.

S4. Castresana, J. (2000). Selection of conserved blocks from multiple alignments for their use in phylogenetic analysis. Molecular biology and evolution *17*, 540-552.

S5. Yang, Z. (2007). PAML 4: Phylogenetic analysis by maximum likelihood. Mol. Biol. Evol. *24*, 1586-1591.

S6. Kyte, J., and Doolittle, R.F. (1982). A simple method for displaying the hydropathic character of a protein. Journal of molecular biology *157*, 105-132.

S7. Benton, M.J., Donoghue, P.C.J., and Asher, R.J. (2009). Calibrating and constraining molecular clocks. In The Timetree of Life, B.S. Hedges and S. Kumar, eds. (Oxford, England: Oxford University Press), pp. 35-86.

S8. Warnock, R.C., Yang, Z., and Donoghue, P.C. (2012). Exploring uncertainty in the calibration of the molecular clock. Biology letters *8*, 156-159.

S9. Benton, M.J., Donoghue, P.C.J., Asher, R.J., Friedman, M., Near, T.J., and Vinther, J. (2015). Constraints on the timescale of animal evolutionary history. Paleontolog. Elect.

S10. Benton, M.J., and Donoghue, P.C.J. (2007). Paleontological evidence to date the tree of life. Mol. Biol. Evol. *24*, 26-53.

S11. Donoghue, P.C., and Benton, M.J. (2007). Rocks and clocks: calibrating the tree of life using fossils and molecules. Trends Ecol. Evol. *22*, 424-431.

S12. Inoue, J., Donoghue, P.C.H., and Yang, Z. (2010). The impact of the representation of fossil calibrations on Bayesian estimation of species divergence times. Syst. Biol. *59*, 74-89.

S13. Yang, Z., and Rannala, B. (2006). Bayesian estimation of species divergence times under a molecular clock using multiple fossil calibrations with soft bounds. Mol. Biol. Evol. *23*, 212-226.

S14. Thorne, J.L., Kishino, H., and Painter, I.S. (1998). Estimating the rate of evolution of the rate of molecular evolution. Mol. Biol. Evol. *15*, 1647-1657.

S15. dos Reis, M., and Yang, Z. (2011). Approximate likelihood calculation for Bayesian estimation of divergence times. Mol. Biol. Evol. *28*, 2161–2172.

S16. Le, S.Q., and Gascuel, O. (2008). An improved general amino acid replacement matrix. Mol. Biol. Evol. *25*, 1307-1320.

S17. Yang, Z. (1994). Maximum likelihood phylogenetic estimation from DNA sequences with variable rates over sites: approximate methods. J. Mol. Evol. *39*, 306-314.

S18. Rannala, B., and Yang, Z. (2007). Inferring speciation times under an episodic molecular clock. Syst. Biol. *56*, 453-466.
S19. dos Reis, M., Zhu, T., and Yang, Z. (2014). The impact of the rate prior on Bayesian estimation of divergence times with multiple Loci. Syst. Biol. *63*, 555-565.
